# The reactions of 2-ethoxymethylidene-3-oxo esters and their analogues with 5-aminotetrazole as a way to novel azaheterocycles

**DOI:** 10.3762/bjoc.11.44

**Published:** 2015-03-23

**Authors:** Marina V Goryaeva, Yanina V Burgart, Marina A Ezhikova, Mikhail I Kodess, Viktor I Saloutin

**Affiliations:** 1 Postovsky Institute of Organic Synthesis, Ural Branch, Russian Academy of Sciences, 22 S. Kovalevskoy St., 620137 Ekaterinburg, Russia

**Keywords:** 5-aminotetrazole, cyclisation, diethyl 2-ethoxymethylidenemalonate, 2-ethoxymethylidene-3-oxo esters, ethyl 2-ethoxymethylidenecyanoacetate

## Abstract

The interaction of 2-ethoxymethylidene-3-oxo esters and their analogues with 5-aminotetrazole is an efficient synthetic approach to novel azaheterocycles. 2-Ethoxymethylidene-3-oxo esters bearing alkyl substituents react with 5-aminotetrazole to form ethyl 2-azido-4-alkylpyrimidine-5-carboxylates which are capable of subsequent nucleophilic substitution. The use of diethyl 2-ethoxymethylidenemalonate in this reaction resulted in ethyl 7-hydroxytetrazolo[1,5-*a*]pyrimidine-6-carboxylate, while ethyl 2-ethoxymethylidenecyanoacetate yielded 5-[2,6-diamino-3,5-bis(ethoxycarbonyl)pyridinium-1-yl]tetrazol-1-ide through an alternative pathway. Ethyl 2-benzoyl-3-ethoxyprop-2-enoate reacted with 5-aminotetrazole by two reaction routes to form ethyl 2-benzoyl-3-(1*H*-tetrazol-5-ylamino)prop-2-enoate and ethyl 7-(1-ethoxy-1,3-dioxo-3-phenylpropan-2-yl)-5-phenyl-4,7-dihydrotetrazolo[1,5-*a*]pyrimidine-6-carboxylate.

## Introduction

2-Ethoxymethylidene-1,3-dicarbonyl compounds are widely recognized as valuable building blocks in designing various open-chain and heterocyclic compounds, including those used in medical practice [1]. The possibility to generate pyrimidine and azolopyrimidine systems based thereon, which exhibit a wide spectrum of biological activity due to structural similarity with nitrogenous bases, is of a special interest [2–5].

Interaction of non-fluorinated 2-ethoxymethylidene-3-oxo esters with aminoazoles either stops at the formation of open-chain 2-(azolylamino)methylidene-3-oxo esters [6] or leads directly to azolo[1,5-*a*]pyrimidines [7–9] depending on the structure of the starting reagents and reaction conditions. We have recently found that the cyclization of polyfluoroalkyl-containing 2-ethoxymethylidene-3-oxo esters with aminoazoles in contrast to non-fluorinated analogues results in the stable dihydroazolo[1,5-*a*]pyrimidines containing a *gem*-aminoalcohol fragment at the polyfluoroalkyl substituent [10–11]. The reactions of ethyl 2-ethoxymethylidene-3-oxo-3-(polyfluoroalkyl)propionates with 3-amino-5-hydroxypyrazole allowed to obtain not only dihydropyrazolo[1,5-*a*]pyrimidines but also pyrazolo[3,4-*b*]pyridines due to the peculiarities of the binucleophile used [12].

To the best of our knowledge, there is no published data about the interaction of 2-ethoxymethylidene-3-oxo esters and their analogues with 5-aminotetrazole (**5-AT**), although these reactions can be used to generate functionalized tetrazolo[1,5-*a*]pyrimidines. Derivatives of these heterocycles are promising objects for biological testing, because substances possessing apoptotic and antitumor [13], antiviral [14], antimicrobial and antioxidant activity [15] have been found among them. The known tetrazolo[1,5-*a*]pyrimidines were synthesized by cyclocondensation of **5-AT** with 1,3-dicarbonyl compounds or their derivatives [16]. Moreover, a convenient method for obtaining this heterocyclic skeleton is the three-component Biginelli condensation of 3-oxo esters, aldehyde and **5-AT** [17–18] or its modification starting from 2-benzylidene-3-oxo esters and **5-AT** [19–20].

It may be expected that the use of **5-AT** as a nucleophile in the reactions with 2-ethoxymethylidene-3-oxo esters will alter the traditional ways of cyclization. Such changes may be caused by lower basicity of **5-AT** (p*K*_b_ = 12.18 [21]) compared with other aminoazoles and the ability of its derivatives to azide–tetrazole isomerism [22–23].

The present paper focuses on studying the peculiarities of interaction of 2-ethoxymethylidene-3-oxo esters **1a–d** and their analogues (diethyl 2-ethoxymethylidenemalonate (**1e**), ethyl 2-ethoxymethylidenecyanoacetate (**1f**)) with **5-AT** as a synthetic approach to various azahetrocycles.

## Results and Discussion

**Scheme 1:**
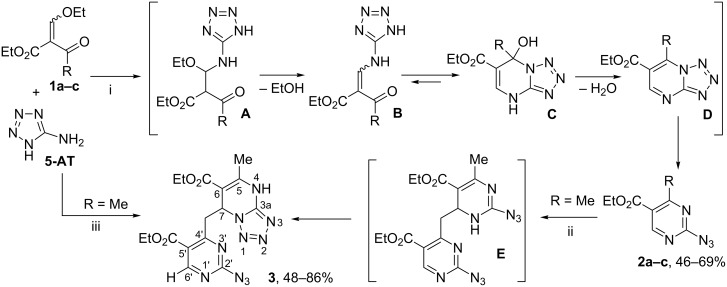
Interaction of 2-ethoxymethylidene-3-oxo esters **1a–c** with **5-AT**. R = CF_3_ (**a**), (CF_2_)_2_H (**b**), Me (**c**). Conditions, i: TFE, Δ, 32–38 h; ii: Air, rt, 12 days; iii: EtOH, rt, 9 days.

It has been found that 2-ethoxymethylidene-3-oxo esters **1a–c** react with **5-AT** in ethanol under reflux to form inseparable mixtures of the products, and while conducting this reaction in refluxing 1,4-dioxane even for a prolonged time (32 h) we observed incomplete conversion of the starting material and low selectivity of transformations. However, 2-azidopyrimidines **2a–c** instead of the expected tetrazolylaminomethylidene-3-oxo esters **В** or tetrazolo[1,5-*a*]pyrimidines **С**, **D** were obtained in 2,2,2-trifluoroethanol (TFE) under reflux (Scheme 1).

Apparently, at first esters **1a–c** interact with **5-AT** at the ethoxymethylidene group to give adduct **A**, which after the elimination of ethanol affords the intermediate tetrazolylaminomethylidene derivative **B**. As a result of regiospecific addition of NH-group to acyl fragment, the intermediate **B** gives dihydrotetrazolo[1,5-*a*]pyrimidine **C**, which is further transformed into tetrazolo[1,5-*a*]pyrimidine **D** after elimination of water. Because of the ability of tetrazole to ring opening at the N1–N8 bond, tetrazolo[1,5-*a*]pyrimidine **D** undergoes azide–tetrazole isomerism to form isomeric 2-azidopyrimidines **2a–c**. The electron-withdrawing substituents in the heterocycle are known to facilitate the opening of the fused tetrazole ring because of decreased electron density at the bridgehead nitrogen atom [24]. Obviously, the ethoxycarbonyl group at C-6 in compounds **2a–c** leads to a significant decrease in the stability of the tetrazole ring and shifts the equilibrium towards the azide isomer.

The azide structure of compounds **2a–c** was confirmed by the presence of absorption bands at ν 2144–2146 cm^–1^, which are typical for the vibration of the azide group in the IR sperctrum [25]. The ^13^C NMR spectra of compounds **2a–c** in CDCl_3_ showed the resonance of the carbon atom C-2 at δ 163.39–163.89 ppm, which is characteristic of the azide form [22–23].

In contrast to polyfluoroalkylated analogues **2a,b**, 4-methyl-2-azidopyrimidine **2c** is not stable and converts into compound **3** during standing on open air for 9 days without a solvent. Compound **3** was obtained also by the reaction of ester **1c** with **5-AT** in ethanol at room temperature; azidopyrimidine **2с** was detected by TLC as an intermediate in this reaction.

The structure of bis-heterocycle **3** was confirmed by ^1^H and ^13^C NMR spectra, including 2D ^1^H,^13^C HSQC/HMBC experiments. So, in the ^13^C NMR spectrum the signal of C-3a carbon of the one heterocyclic fragment was observed at δ 149.71 ppm, which is characteristic of the bridgehead carbon atom in 4,7-dihydrotetrazolopyrimidines [20]; and the signal of C-2' carbon of another hetaryl moiety was observed at δ 163.41 ppm, which is typical for the C-2 carbon in 2-azidopyrimidines **2a–c**. The HMBC cross-peaks between NH proton (δ 10.7 ppm) and carbons of the methyl group (δ 19.42 ppm), C-6 (δ 97.77 ppm) and C-3a (δ 149.71 ppm) in tetrazolopyrimidine fragment, as well as cross-peaks between H-6’ proton (δ 9.02 ppm) and C-5’ (δ 120.17 ppm) and C-2’ (δ 163.41 ppm) carbons in azidopyrimidine system supported structure **3**.

The formation of bis-heterocycle **3** is due to the unique structure of the starting 2-azidopyrimidine **2c** bearing the nucleophilic Me group and the electrophilic CH centre. Evidently, Me group of pyrimidine **2c** reacts with CH-active centre of the another pyrimidine molecule through the formation of intermediate **E** followed by azido-tetrazole rearrangement.

**Scheme 2:**
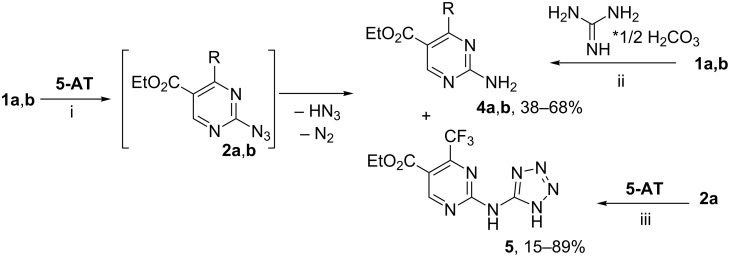
Synthesis of pyrimidines **4a**,**b** and **5**. R= CF_3_ (**a**), (CF_2_)_2_H (**b**). Conditions: i: 1,4-dioxane, NaOAc, Δ, 16–18 h; ii DMF, NaOAc, 80 °C, 24–26 h; iii: 1,4-dioxane, Et_3_N, Δ, 40 h.

In addition, we have found that the use of catalytic amounts of sodium acetate in the reaction of fluorinated esters **1a,b** with **5-AT** in 1,4-dioxane under reflux leads to the formation of ethyl 2-amino-4-(polyfluoroalkyl)pyrimidine-5-carboxylates **4a,b** (Scheme 2). In the case of **1a** (R = CF_3_), ethyl 2-(1*H*-tetrazol-5-ylamino)-4-(trifluoromethyl)-pyrimidine-5-carboxylate (**5**) was isolated as a by-product in the reaction of ester **1a** and **5-AT** (Scheme 2).

Apparently, in this reaction azidopyrimidines **2a**,**b** acted as intermediates that were decomposed under heating in 1,4-dioxane with elimination of nitrogen and nitrenes [26] (Scheme 2). The latter can cleave a hydrogen atom from the other substrates (in our case, from **5-AT**) to yield 2-aminopyrimidines **4a**,**b**.

In addition, the counter synthesis of pyrimidines **4a**,**b** was realized by the reaction of esters **1a**,**b** with guanidine carbonate under similar conditions (Scheme 2). Earlier, pyrimidine **4a** that exhibited antiviral activity against HSV-1 was obtained by cyclization of ethyl 2-dimethylaminomethyliden-4,4,4-trifluoroacetoacetate [27].

The formation of 2-(tetrazolylamino)pyrimidine **5** is the result of nucleophilic substitution of the azide group in intermediate **2a** by the aminotetrazole fragment, as the azide group is a good nucleofuge [28]. Indeed, the reaction of azidopyrimidine **2a** with **5-AT** made it possible to obtain 2-(tetrazolylamino)pyrimidine **5** in good yield (Scheme 2).

**Figure 1:**
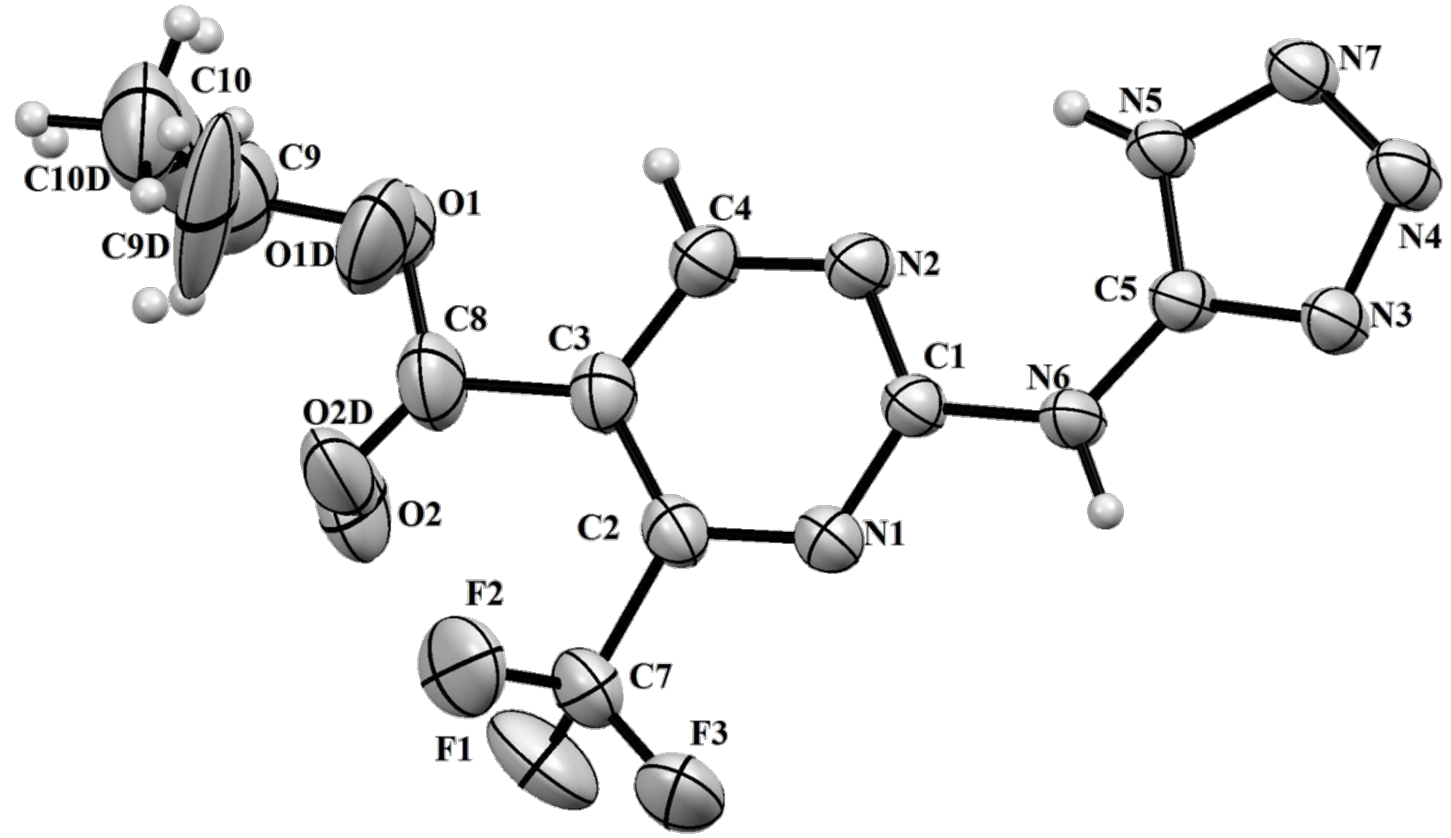
X-ray crystal structure of compound **5** (ORTEP drawing, 50% probability level).

The structure of pyrimidine **5** was confirmed by X-ray diffraction analysis (Figure 1).

**Scheme 3:**
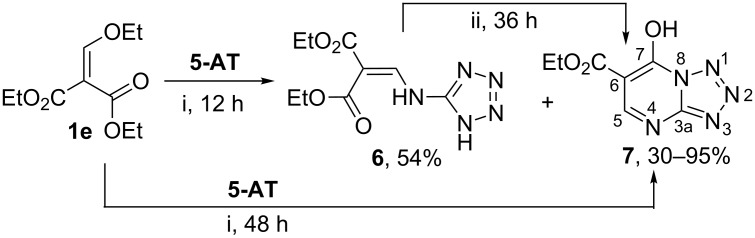
Interaction of 2-ethoxymethylidene malonate **1e** with **5-AT**. Conditions: i: EtOH, Et_3_N, Δ; ii: EtOH, Δ.

Further in this reaction we used 2-ethoxymethylidenemalonate (**1e**) as an analogue of 3-oxo esters having the ester fragment instead of an acyl group. So the interaction of diester **1e** with **5-AT** in refluxing ethanol in the presence of catalytic amounts of triethylamine proceeded in a classical way and resulted in 2-tetrazolylaminomethylidene malonate **6** that was cyclised in ethyl-7-hydroxytetrazolo[1,5-*a*]pyrimidine-6-carboxylate (**7**) (Scheme 3). The most suitable condition for the formation of heterocycle **7** was a prolonged refluxing in ethanol in the presence of triethylamine. In the absence of catalyst, the complete conversion of diester **1e** into amino derivative **6** was not observed even after refluxing for 48 h. Cyclization of compound **6** into tetrazolopyrimidine **7** was performed under prolonged heating without the base.

The structure of tetrazolo[1,5-*a*]pyrimidine **7** was confirmed by IR, ^1^H, ^13^C, 2D ^1^H,^13^C and 2D ^1^H,^15^N HMBC NMR spectra. Thus, the IR spectrum of compound **7** in the solid state did not have the absorption band of the azide group at ν 2100–2200 cm^−1^, but contained the absorption bands of hydroxy groups O–H (ν 3419 cm^−1^) and carbonyl fragments C=O (ν 1715 cm^-1^). The ^1^H and ^13^C NMR spectra in DMSO-*d*_6_ contained one set of the signals corresponding to the tetrazole form with a resonance of C-3a at δ 158.63 ppm. Two low-field carbons C-7 and C-3a (δ_C_ 153.48 and 158.63 ppm) in the ^13^C NMR spectrum cannot be distinguished by HMBC experiment, since both give cross-peaks with the proton H-5 due to the constant ^3^*J*_C,H_ through three bonds. However, the value of the constant for bridgehead carbon C-3a should be greater than that for C-7 [29–30]. The values of the spin–spin coupling constants obtained from the proton-coupled ^13^C NMR spectrum (^3^*J*_C-3a,H-5_ = 16.6 Hz, ^3^*J*_C-7,H-5_ = 7.6 Hz) enable us to assign the shift at δ 158.63 ppm to the C-3a carbon.

We carried out a series of 2D ^1^H,^15^N HMBC experiments for compound **7** varying evolution delay time. In the HMBC spectra correlations between the CH proton (δ_H_ = 8.7 ppm) and three ^15^N atoms at δ_N_ = 210.6, 247.4, 306.3 ppm were observed. Based on the published data [22,31], we have assigned these chemical shifts to nitrogens N-4, N-8, and N-3, respectively, in the structure **7**. In the HMBC spectrum of an alternative isomeric structure of ethyl-5-hydroxytetrazolo[1,5-*a*]pyrimidine-6-carboxylate formed as a result of tetrazole rearrangement, it should be expected the intense correlation between CH-proton and tetrazole nitrogen with the chemical shift in the range of 350–400 ppm. However such correlation did not appear in HMBC experiments, which allows us to exclude this structure from consideration.

We have found that replacing of the alkyl group by the phenyl residue in 3-oxo ester affects the reaction with **5-AT**. So the reaction of ethyl 2-benzoyl-3-ethoxyprop-2-enoate (**1d**) with **5-AT** in ethanol under reflux led to a complex mixture of products. Separation of the reaction mixture by column chromatography afforded only ethyl benzoylacetate (**8**) that is likely to be formed as a result of decomposition of starting ester **1d**. The decomposition of 2-alkoxymethylidene-containing 3-oxo esters and their analogues to the corresponding esters in the reactions with N- and C-nucleophiles is known [32–33].

**Scheme 4:**
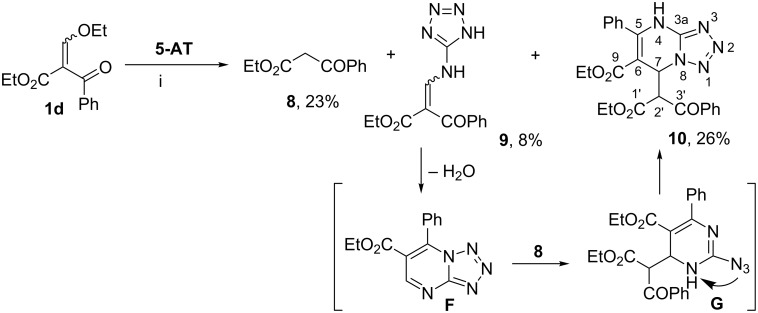
The reaction of 3-oxo ester **1d** with **5-AT**. Conditions, i: TFE, Δ, 48 h.

When this reaction was carried out in refluxing TFE, we also obtained a mixture of products. However, in this case we managed to isolate tetrazolylaminomethylidene derivative **9** and compound **10** in addition to ester **8** (Scheme 4).

Ethyl 2-benzoyl-3-(1*H*-tetrazol-5-ylamino)prop-2-enoate (**9**) exists as a mixture of *Z-*and *E-*isomers stabilized by the intramolecular H-bond in contrast to the symmetrical malonate derivative **6**.

The structure of compound **10** was established by ^1^H and ^13^C NMR including 2D ^1^H,^13^C HSQC/HMBC spectra. According to the 1D NMR spectra, compound **10** has two ethoxycarbonyl groups, two phenyl moieties, two sp^3^-C atoms of methine group with vicinal coupling constant *J*_H2'-H7_ = 2.8 Hz, and NH-group (δ 10.48 ppm). The protonated carbons of **10** were directly assigned through the ^1^*J*_CH_ connectivities provided by the HSQC experiment. The most informative cross-peaks of HMBC spectrum in CDCl_3_ for compound **10** were as follows: H-2'→C-1', C-3'; H-2'→C-6, C-7; H-7→C-5, C-6, C-9, and C-1'; NH→C-6, C-3a, C*_ipso_*. The carbonyl carbons were identified by long-range connectivities with protons OCH_2_ (C-9 and C-1') and H*_ortho_* (C-3').

It can be assumed the formation of heterocycle **10** proceeds via tetrazolopyrimidine intermediate **F** (Scheme 4). The mass spectrum of amino derivative **9** shows the possibility of tetrazolopyrimidine **F** formation since it has the peak of (*m*/*z* 269 [M − H_2_O]^+^). Then tetrazolopyrimidine **F** adds benzoylacetic ester (**8**) as a *C*-nucleophile at the electrophilic CH-center. The reaction is accompanied by tetrazole rearrangement through the formation of intermediate **G** to give heterocycle **10**. Compound **10** was isolated as one diastereomer, but the relative configuration of its stereogenic centers was not determined.

**Scheme 5:**
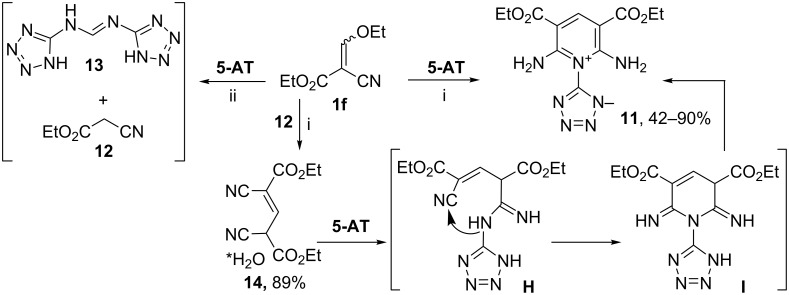
Interaction of ester **1f** with **5-AT**. Conditions i: EtOH (or TFE), Et_3_N, Δ, 40–60 min; ii: AcOH (or EtOH, AcOH), Δ, 32 h.

Moreover, we studied the reaction of ethyl 2-ethoxymethylidenecyanoacetate (**1f**) with **5-AT**. It has been found that this reaction does not occur either in refluxing ethanol or in refluxing TFE, but addition of catalytic amounts of triethylamine resulted in compound **11** in good yield (Scheme 5).

**Figure 2:**
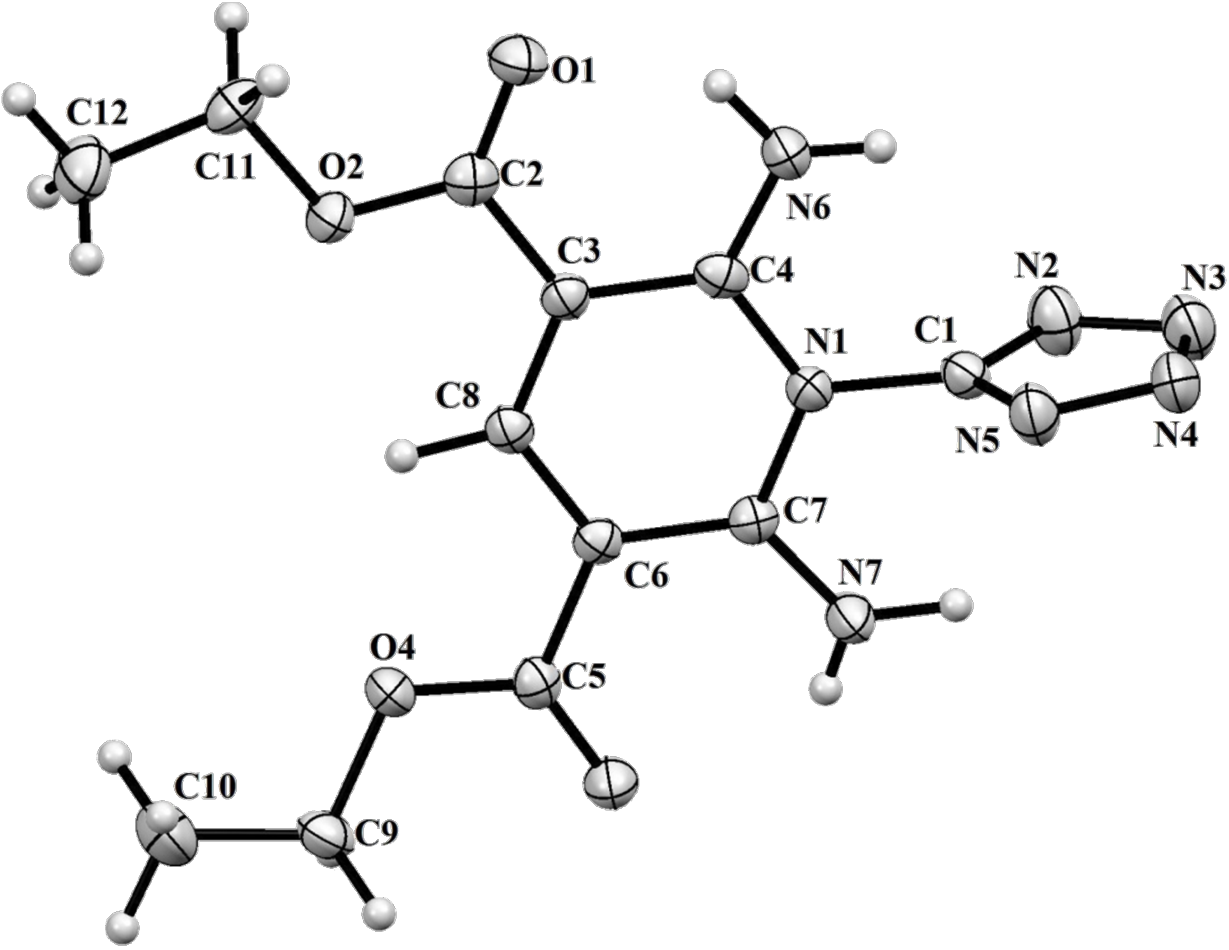
X-ray crystal structure of compound **11** (ORTEP drawing, 50% probability level).

The IR spectrum of compound **11** has strong absorption bands of the amino group (ν 3292, 3238 cm^−1^) and the carbonyl substituent (ν 1699 cm^−1^). The ^1^H NMR spectral data allowed us to conclude that the molecule of compound **11** is symmetric and bears one methine proton, two amino groups and two ethoxycarbonyl substituents. The final structure of compound **11** as 5-[2,6-diamino-3,5-bis(ethoxycarbonyl)pyridinium-1-yl]tetrazol-1-ide was confirmed by X-ray diffraction (Figure 2).

The preparation of compound **11** in the reaction of ester **1f** with **5-AT** was unexpected, because it is known that ester **1f** interacts with 3-amino-1,2,4-triazole in refluxing ethanol [8] and with substituted 5-amino-1,2,4-triazoles in acetic acid [34] to afford triazolo[1,5-*a*]pyrimidines. However, we did not observe the complete conversion of ester **1f** in the reaction with **5-AT** even under prolonged reflux in acetic acid. According to GC–MS data, the reaction mixture contained cyanoacetic ester (**12**) (*m*/*z* 113 [М]^+^) and ethyl 3-(1*H*-tetrazol-5-ylamino)prop-2-enoate (**13**) (*m*/*z* 182 [М + 2]^+^) along with ester **1f** (*m*/*z* 169 [М]^+^) (Scheme 5).

It can be assumed that ethyl cyanoacetate (**12**), formed as a result of ethoxymethylidene group migration to **5-AT**, reacts with ester **1f** to give intermediate diester **14** (Scheme 5). The latter is cyclised with **5-AT** into pyridine **11** via intermediates **H** and **I**. We carried out the three-component reaction of ester **1f**, cyanoacetic ester **12** and **5-AT** in the presence of triethylamine to confirm the proposed mechanism. The product **11** was obtained in excellent yield. Moreover, we managed to isolate diethyl 2,4-dicyanopent-2-enedioate (**14**, Scheme 5), which was previously obtained as a sodium salt [35].

## Conclusion

In summary, we can conclude that 2-ethoxymethylidene-substituted esters **1a–f** show different ability to react with **5-AT** depending on their structure. Besides, these conversions differ from known cyclisations with other aminoazoles [7–12]. These differences can be explained by the properties of **5-AT**. Thus, due to the tendency of the tetrazole ring towards isomerisation to an azide group, 3-oxo esters **1a–c** with alkyl substituents form 2-azidopyrimidines **2a–c** that are capable for further modification at the electrophilic centre C-6 and the azide group. Another difference is the weak basicity of **5-AT**, which requires the basic catalysis in the reaction of 2-ethoxymethylidenecyanoacetate **1f** with **5-AT**. This resulted in an alternative reaction pathway with a partial decomposition of the starting ester **1f** to cyanoacetic ester **12** and subsequent formation of pyridine **11**.

Thus, we have found an effective and simple reaction for the preparation of substituted pyrimidine and pyridine derivatives. The resulting compounds are important pharmacophores or may be used as a starting material for synthesis of other functionalized pyrimidines through nucleophilic substitution.

## Supporting Information

File 1 Experimental section and the copies of all ^1^H, ^13^C, and ^19^F NMR spectra of all new compounds.
