# Supplementary material for: The reactions of 2-ethoxymethylidene-3-oxo esters and their analogues with 5-aminotetrazole as a way to novel azaheterocycles
Source: Beilstein J Org Chem. 2015 Mar 23;11:385–91. doi: 10.3762/bjoc.11.44 (PMC4419505; doi:10.3762/bjoc.11.44)
Supplement: File 1 — Experimental section and the copies of all 1H, 13C, and 19F NMR spectra of all new compounds. [file Beilstein_J_Org_Chem-11-385-s001.pdf]

Supporting Information

for

The reactions of 2-ethoxymethylidene-3-oxo esters and their analogues with 5-aminotetrazole as a way to novel azaheterocycles

Marina V. Goryaeva*, Yanina V. Burgart, Marina A. Ezhikova, Mikhail I. Kodess and Viktor I. Saloutin

Address: Postovsky Institute of Organic Synthesis, Ural Branch, Russian Academy of Sciences, 22 S. Kovalevskoy St., 620137 Ekaterinburg, Russia

Email: Marina V. Goryaeva* - pmv@ios.uran.ru

* Corresponding author

Experimental section and the copies of all ^{1}H, ^{13}C, and ^{19}F NMR spectra of all new compounds.

Table of contents:

Pages S2–S10: Detailed experimental procedures and spectroscopic data for all synthesized compounds.

Page S12: References

Pages S13–S44: Copies of ^{1}H, ^{19}F and ^{13}C NMR spectra of new compounds.

Detailed experimental procedures and spectroscopic data for all synthesized compounds.

Experimental

General: Melting points were measured in open capillaries on a Stuart SMP3 melting point apparatus. The diffuse reflection spectra (DRIFT) were recorded on a Perkin Elmer Spectrum One Fourier transform infrared spectrometer, the attenuated total reflectance spectra (ATR) were registered on Nicolet 6700 FT-IR spectrometer in the range from 400 to 4000 cm^{-1}. The ^1H, ^{19}F and ^{13}C NMR spectra were recorded on Bruker DRX-400 and AVANCE-500 instruments using Me_4Si as an internal standard for ^1H, and C_6F_6 for ^{19}F. The ^{13}C chemical shifts were measured from the solvent signal DMSO-d_6 (δ_C 39.5 ppm) or $CDCl_3$ (δ_C 77.0 ppm). All signals in the ^1H and ^{13}C NMR spectra were assigned on the basis of 2D $^1H,^{13}C$ HSQC and HMBC experiments. Mass spectra of **2a,b,c** and **4b** were obtained using GC-MS Trace GC Ultra DSQ II with capillary column Termo TR-5ms (30 m x 0.25 mm x 0.25 μm), mass spectra of compounds **3**, **5-*7***, **9–11, 14** were performed using a Shimadzu GCMS-QP2010 Ultra. The microanalyses were carried out on a Perkin Elmer PE 2400 series II elemental analyzer. The column chromatography was performed on Merck silica gel 60 (0.063–0.200 mm).

Ethyl 2-ethoxymethylidene-3-oxo-3-(polyfluoroalkyl)propionates (**1a,b**) [1], ethyl 2-ethoxymethylideneacetoacetate (**1c**) and ethyl 2-ethoxymethylidenecyanoacetate (**1f**) [2], ethyl 2-benzoyl-3-ethoxyprop-2-enoate (**1d**) [3] were obtained according to the literature procedures. Diethyl 2-ethoxymethylidenemalonate (**1e**) and 5-aminotetrazole monohydrate are commercially available (purchased from Alfa Aesar and Sigma-Aldrich respectively).

General procedure for the synthesis of compounds 2a-c: A mixture of the corresponding ester **1a-c** (3 mmol) and 5-aminotetrazole (0.31 g, 3 mmol) in trifluoroethanol (20 ml) was refluxed for 32-38 h. After the completion of the reaction (TLC monitoring), the reaction mixture was evaporated to dryness under reduced pressure, the residue was purified by column chromatography (chloroform as an eluent) to afford compounds **2a-c**.

Ethyl 2-azido-4-(trifluoromethyl)pyrimidine-5-carboxylate (2a): Yellow oil; yield 0.54 g (69%); ATR (cm^{-1}) ν 2988 (C–H), 2144 (N=N=N), 1731 (C=O), 1581, 1551 (C=C–C=N), 1195–1084 (C-F); ^{1}H NMR (500 MHz, $CDCl_3$) δ 1.41 (t, J = 7.1 Hz, 3H, CH_3), 4.44 (q, J = 7.1 Hz, 2H, OCH_2), 9.13 (s, 1H, H–6); ^{19}F NMR (470 MHz, $CDCl_3$) δ 95.34 (s, CF_3); ^{13}C NMR (126 MHz, $CDCl_3$) δ 13.81 (CH_3), 62.90 (OCH_2), 119.57 (q, $^1J_{C\text{-}F}$ = 276.6 Hz, CF_3), 119.67 (q, $^3J_{C\text{-}F}$ = 0.9 Hz, C-5), 155.79 (q, $^2J_{C\text{-}F}$ = 37.8 Hz, C-4), 162.54 (COO), 162.97 (C-6), 163.89 (q, $^4J_{C\text{-}F}$ = 1.2 Hz, C-2); MS (EI) m/z (%): 261 (M^+, 20), 233 (37), 216 (33), 188 (22), 162 (17), 148 (6), 133 (10), 120 (17), 106 (11), 92 (34), 69 (81), 53 (28), 29 (100); Anal calcd for $C_8H_6F_3N_5O_2$: C, 36.79; H, 2.32; N, 26.82; found: C, 36.83; H, 2.31; N, 26.81.

Ethyl 2-azido-4-(1,1,2,2-tetrafluoroethyl)pyrimidine-5-carboxylate (2b): Yellow oil, yield 0.53 g (60%); ATR (cm^{-1}) ν 2989, 2940 (C–H), 2146 (N=N=N), 1732 (C=O), 1581, 1547 (C=C–C=N), 1130–1102 (C-F); ^{1}H NMR (400 MHz, $CDCl_3$) δ 1.41 (t, J = 7.1 Hz, 3H, CH_3), 4.44 (q, J = 7.1 Hz, 2H, OCH_2), 6.61 (tt, $J_{H\text{-}F}$ = 52.9, 5.7 Hz, 1H, $(CF_2)_2H$), 9.03 (s, 1H, H-6); ^{19}F NMR (375 MHz, $CDCl_3$) δ 23.76 (dm, $J_{F\text{-}H}$ = 52.9 Hz, 2F, CF_2H), 44.54 (m, 2F, CF_2); ^{13}C NMR (126 MHz, $CDCl_3$) δ 13.89 (CH_3), 63.02 (OCH_2), 109.38 (tt, $J_{C\text{-}F}$ = 252.0, 31.3 Hz, CF_2H) 112.19 (tt, $J_{C\text{-}F}$ = 255.1, 26.7 Hz, CF_2), 121.07 (C-5), 158.17 (t, $^2J_{C\text{-}F}$ = 28.4 Hz, C-4), 162.17 (s, C-6), 163.31 (COO), 163.39 (t, $^4J_{C\text{-}F}$ = 1.5 Hz, C-2); MS (EI) m/z (%): 293 (M^+, 20), 265 (17), 248 (36), 220 (23), 209 (14), 171 (9), 142

(26), 120 (21), 101 (64), 92 (46), 53 (34), 51 (58), 29 (100); Anal; calcd for $C_9H_7F_4N_5O_2$: C, 36.87; H, 2.41; N, 23.89; found: C, 36.81; H, 2.40; N, 23.85.

Ethyl 2-azido-4-methylpyrimidine-5-carboxylate (2c): Yellow oil, yield 0.29 g (46%); ATR (cm^{-1}) ν 2984 (C–H), 2144 (N=N=N), 1725 (C=O), 1576, 1544 (C=C–C=N); ^1H NMR (500 MHz, $CDCl_3$) δ 1.41 (t, J = 7.1 Hz, 3H, CH_3), 2.80 (s, Me), 4.40 (q, J = 7.1 Hz, 2H, OCH_2), 9.03 (s, 1H, H–6); ^{13}C NMR (126 MHz, $CDCl_3$) δ 14.16 (CH_3), 24.42 (CH_3C-4), 61.48 (OCH_2), 119.30 (C-5), 161.31 (C-6), 163.49 (C-2), 164.21 (COO), 171.87 (C-4); MS (EI) m/z (%): 207 (M^+, 53), 162 (31), 134 (43), 109 (60), 67 (100), 53 (57), 43 (91), 29 (89); Anal calcd for $C_8H_9N_5O_2$: C, 46.38; H, 4.38; N, 33.80; found: C, 46.34; H, 4.22; N, 33.83.

Ethyl 7-{[2-azido-5-(ethoxycarbonyl)pyrimidin-4-yl]methyl}-5-methyl-4,7-dihydro-tetrazolo[1,5-*a*]pyrimidine-6-carboxylate (3):

Method A. A mixture of ethyl 2-ethoxymethyleneacetoacetate (**1c**) (0.56 g, 3 mmol) and 5-aminotetrazole (0.31 g, 3 mmol) in EtOH (20 ml) was stirred at room temperature for 9 days. After the completion of the reaction (TLC monitoring), the reaction mixture was evaporated to dryness under reduced pressure, the residue was washed with diethyl ether and crystallized from hexane to give 0.60 g (48%) of compound **3**. **Method B**. Oily azidopyrimidine 2c (0.30 g, 0.7 mmol) solidified at the room temperature for 10-12 days. Then the resulting solid was washed with diethyl ether and crystallized from hexane to afford 0.26 g (86%) of compound **3** as yellow powder, mp 145–146 °C; DRIFT (cm^{-1}) ν 3325 (N–H), 2982 (C–H), 2137 (N=N=N), 1705 (C=O), 1577, 1541 (C=C-C=N); ^1H NMR (500 MHz, $CDCl_3$) δ 1.37 (m, 6H, CH_3 and CH_3'), 2.55 (s, 3H, Me), 3.47 (dd, J = 13.4, 3.9 Hz, 1H, CH^B), 4.16 (dd, J = 13.4, 7.0 Hz, 1H, CH^A), 4.26 (q, J = 7.2 Hz, 2H, OCH_2), 4.24–4.35 (m, 2H, OCH_2), 6.34 (dd, J = 7.0, 3.9 Hz, 1H, H-7), 9.02 (s, 1H, H-6'), 10.72 (s, 1H, NH); ^{13}C NMR (126 MHz, $CDCl_3$) δ 14.10 (CH_3), 14.24 (CH_3), 19.42 (CH_3C-5), 41.01 (CH_2), 55.28 (C-7), 60.73 (OCH_2), 61.84 (OCH_2'), 97.77 (C-6), 120.17 (C-5'),

147.83 (C-5), 149.71 (C-3a), 161.79 (C-6'), 163.41 (C-2'), 163.69 (COO), 164.71 (COO), 169.09 (C-4'); MS (EI) m/z (%): 207 ($[M / 2]^+$, 69), 179 (3), 162 (27), 134 (48), 109 (53), 80 (45), 67 (73), 43 (100); Anal calcd for $C_{16}H_{18}N_{10}O_4$: C, 46.38; H, 4.38; N, 33.80; found: C, 46.35; H, 4.39; N, 33.76.

General procedures for the synthesis of compounds 4a,b: Method A. A mixture of the corresponding ester **1a,b** (3 mmol), 5-aminotetrazole (0.31 g, 3 mmol) and sodium acetate trihydrate (0.20 g, 1.5 mmol) in 1,4-dioxane (25 ml) was refluxed for 16-18 h. After the completion of the reaction (TLC monitoring), the reaction mixture was poured into cool water (100 ml). The resulting precipitate was filtered off, washed with diethyl ether and crystallized from ethanol to give compounds **4a,b**. The filtrate and washing solutions after purification of compound 4a were used to obtain compound **5**. **Method B.** A mixture of the corresponding ester 1a,b (3 mmol), guanidine carbonate (0.27 g, 3 mmol) and sodium acetate trihydrate (0.40 g, 3 mmol) in dimethylformamide (15 ml) was stirred under heating (80 °C) for 24–26 h. After the completion of the reaction (TLC monitoring), the reaction mixture was poured into cool water (200 ml). The resulting precipitate was filtered off and crystallized from ethanol to afford compounds **4a,b**.

Ethyl 2-amino-4-(trifluoromethyl)pyrimidine-5-carboxylate (4a): Yellow powder, yield (method A) 0.27 g (38%), (method B) 0.42 g (60%), mp 178–179 °C. 1H NMR spectrum is identical to that published in [4].

Ethyl 2-amino-4-(1,1,2,2-tetrafluoroethyl)pyrimidine-5-carboxylate (4b): Yellow powder; yields (method A) 0.35 g (44%), (method B) 0.55 g (68%); mp 141–142 °C; DRIFT (cm^{-1}) ν 3372, 3336, 3212 (NH, NH_2), 1716 (C=O), 1666, 1590, 1543 (C=C–C=N), 1179–1069 (C–F); ^1H NMR (500 MHz, $CDCl_3$) δ 1.39 (t, J = 7.2 Hz, 3H, CH_3), 4.37 (q, J = 7.2 Hz, 2H, OCH_2), 5.80 (br. s, 2H, NH_2), 6.68 (tt, J_{H-F} = 53.4, 5.8 Hz, 1H, $(CF_2)_2H$), 8.83 (s, 1H, H-6); ^{19}F NMR (470 MHz, $CDCl_3$) δ 23.46 (dm, J_{F-H} = 53.4 Hz, 2F, CF_2H), 43.43 (m, 2F, CF_2); ^{13}C NMR (126 MHz, $CDCl_3$) δ 14.01 (CH_3), 62.08 (OCH_2),

109.71 (tt, $J_{C\text{-}F}$ = 251.6, 30.8 Hz, CF_2H), 112.52 (tt, $J_{C\text{-}F}$ = 254.4, 26.1 Hz, CF_2), 114.99 (C-5), 158.24 (t, $^2J_{C\text{-}F}$ = 26.9 Hz, C-4), 162.32 (C-6), 162.80 (unres. m, C-2), 164.14 (COO); MS (EI) m/z (%): 267 (M^+, 16), 239 (13), 222 (100), 202 (11), 171 (11), 120 (10), 101 (4), 93 (7), 68 (7), 53 (6), 29 (5); Anal calcd for $C_9H_9F_4N_3O_2$: 40.46; H, 3.40; N, 15.73; found: C, 40.52; H, 3.41; N, 15.75.

Ethyl 2-(1*H*-tetrazol-5-ylamino)-4-(trifluoromethyl)pyrimidine-5-carboxylate (5): Method A. The filtrates after purification of compound **4a** (method A) were combined and evaporated. The resulting precipitate was washed with diethyl ether and crystallized from ethanol to give 0.14 g (15%) of compound **5**. **Method B.** A mixture of 2-azidopyrimidine **2c** (0.5 mmol), 5-aminotetrazole (0.05 g, 0.5 mmol) and triethylamine (3 drops) in 1,4-dioxane (15 ml) was refluxed for 5 days. After the completion of the reaction (TLC monitoring), the reaction mixture was poured into cool water (200 ml). The resulting precipitate was filtered off and crystallized from ethanol to give 0.13 g (89%) of compound **5** as a white powder; mp 216–218 °C; DRIFT (cm^{-1}) ν 3252, 3170 (NH), 2967, 2931 (C–H), 1744 (C=O), 1629, 1583, 1551 (C=C–C=N), 1205–1142 (C–F); ^{1}H NMR (400 MHz, DMSO-d_6) δ 1.33 (t, J = 7.1 Hz, 3H, CH_3), 4.36 (q, J = 7.1 Hz, 2H, OCH_2), 9.12 (s, 1H, H-6), 12.50 (br. s, 1H, NH), 15.81 (br. s, 1H, NH-1'); ^{19}F NMR (375 MHz, DMSO-d_6) δ 97.20 (s, CF_3); ^{13}C NMR (126 MHz, DMSO-d_6) δ 13.71 (CH_3), 61.98 (OCH_2), 115.89 (C-5), 119.85 (q, $^1J_{C\text{-}F}$ = 276.4 Hz, CF_3), 150.78 (br. s, C-5'), 154.00 (q, $^2J_{C\text{-}F}$ = 36.4 Hz, C4), 158.36 (C-2), 162.37 (C-6), 162.53 (COO); MS (EI) m/z (%): 303 (M^+, 8), 275 (3), 258 (16), 247 (22), 220 (100), 192 (34), 152 (22), 124 (18), 77 (22), 69 (14), 53 (14), 45 (8); Anal. Calcd for $C_9H_8F_3N_7O_2$: C, 35.65; H, 2.66; N, 32.34; found: C, 35.62; H, 2.60; N, 32.25.

General procedures for the synthesis of compounds 6, 7: Method A. A mixture of diethyl 2-ethoxymethylenemalonate (**1e**) (0.65 g, 3 mmol), 5-aminotetrazole (0.31 g, 3 mmol) and triethylamine (3 drops) in ethanol (25 ml) was refluxed for 12 h. After the

completion of the reaction (TLC monitoring), the reaction mixture was evaporated to dryness under reduced pressure, the residue was triturated with chloroform and filtered off. The precipitate was crystallized from ethanol to give compound **7**. The filtrate was evaporated, and the residue was crystallized from acetonitrile to yield compound **6**. **Method B.** A mixture of diethyl 2-ethoxymethylenemalonate (**1e**) (0.65 g, 3 mmol), 5-aminotetrazole (0.26 g, 3 mmol) and triethylamine (3 drops) in ethanol (25 ml) was refluxed for 48 h. After the completion of the reaction (TLC monitoring), the reaction mixture was evaporated to dryness under reduced pressure, the residue was crystallized from ethanol to give 0.48 g (76%) of compound **7**.

Method C. The compound **6** (0.13 g, 0.5 mmol) in ethanol (15 ml) was refluxed for 36 h. After the completion of the reaction (TLC monitoring), the reaction mixture was cooled in refrigerator, the precipitate was filtered off to give **7.**

Diethyl [(1*H*-tetrazol-5-ylamino)methylidene]propanedioate (6): White powder; yield (method A) 0.41 g (54%); mp 162–164 °C; DRIFT (cm^{-1}) ν 3300, 3233 (NH), 2996, 2942 (C–H), 1702, 1649 (C=O), 1605, 1567 (C=C, C=N); ^{1}H NMR (400 MHz, DMSO-d_6) δ 1.24 (t, J = 7.1 Hz, 3H, CH_3), 1.27 (t, J = 7.1 Hz, 3H, CH_3), 4.16 (q, J = 7.1 Hz, 2H, OCH_2), 4.24 (q, J = 7.1 Hz, 2H, OCH_2), 8.44 (d, J = 13.0 Hz, 1H, CH), 11.01 (d, J = 13.0 Hz, 1H, NH); ^{13}C NMR (126 MHz, DMSO-d_6) δ 14.02 (CH_3), 14.13 (CH_3), 60.05 (OCH_2), 60.34 (OCH_2), 98.43(C-2), 148.70 (CH), 155.57 (br. s, C-5'), 164.02 (COO), 166.05 (COO); MS (EI) m/z (%): 255 (M^+, 12), 227 (8), 210 (21), 198 (76), 152 (100), 124 (62), 85 (13), 53 (86); Anal calcd for $C_9H_{13}N_5O_4$: C, 42.35; H, 5.13; N, 27.44; found: C, 42.27; H, 5.12; N, 27.38.

Ethyl 7-hydroxytetrazolo[1,5-*a*]pyrimidine-6-carboxylate (7): White powder; yield (method A) 0.19 g (30%), (method B) 0.48 g (76%), (method C) 0.10 g (95%); mp 236–238 °C; DRIFT (cm^{-1}) ν 3419 (O–H), 1715 (C=O), 1633, 1534 (C=C–C=N); ^{1}H NMR (500 MHz, DMSO-d_6) δ 1.28 (t, J = 7.1 Hz, 3H, CH_3), 3.36 (s, OH + H_2O solv.), 4.21 (q,

J = 7.1 Hz, 2H, OCH_2), 8.71 (s, 1H, H-5); ^{13}C NMR (126 MHz, DMSO-d_6) δ 14.39 (qt, *J* = 126.6, 2.6 Hz, CH_3), 59.14 (tq, *J* = 147.1, 4.5 Hz, OCH_2), 98.15 (d, *J* = 6.2 Hz, C-6), 153.48 (d, $^3J_{C7\text{-}H5}$ = 7.6 Hz, C-7), 158.63 (d, $^3J_{C3a\text{-}H5}$ = 16.6 Hz, C-3a), 160.08 (d, *J* = 177.6 Hz, C-5), 165.39 (q, *J* = 3.2 Hz, COO); MS (EI) m/z: 209 (M^+, 9), 192 (30), 165 (40), 137 (70), 124 (28), 109 (36), 95 (22), 81 (28), 69 (46), 53 (100), 44 (38), 43 (33). Anal calcd for $C_7H_7N_5O_3$: C, 40.20; H, 3.37; N, 33.48; found: C, 39.99; H, 3.39; N, 33.30.

General procedure for the synthesis of compounds 9, 10: A mixture of ethyl 2-benzoyl-3-ethoxyprop-2-enoate (**1d**) (0.65 g, 3 mmol) and 5-aminotetrazole (0.31 g, 3 mmol) in trifluoroethanol (25 ml) was refluxed for 48 h. After the completion of the reaction (TLC monitoring), the reaction mixture was evaporated to dryness under reduced pressure, the residue was subjected to column chromatography (chloroform as an eluent) to afford ester **8** as the fastest eluting substance, then compound **9**, and compound **10** as a slow eluting substance. The fraction containing compound **9** was evaporated to dryness, the residue was washed with diethyl ether to give compound **9**. The last fraction containing compound **10** was evaporated to dryness, the residue was crystallized from acetonitrile to afford compound **10**.

Ethyl 2-benzoyl-3-(1*H*-tetrazol-5-ylamino)prop-2-enoate (9): White powder; yield 0.07 g (8%); mp 122-124 °C; DRIFT (cm^{-1}) ν 3493, 3007 (N–H, O–H), 3056, 3031, 2980 (C–H), 1663 (C=O, br), 1612, 1584 (C=C); ^{1}H NMR (500 MHz, DMSO-d_6) δ *E* (60%): 0.97 (t, *J* = 7.1 Hz, 3H, CH_3), 3.97 (q, *J* = 7.1 Hz, 2H, OCH_2), 7.39-7.58 (m, 5H, Ph), 8.73 (d, *J* = 13.6 Hz, 1H, H-3), 11.86 (d, *J* = 13.6 Hz, 1H, NH), *Z* (40%): 0.92 (t, *J* = 7.2 Hz, 3H, CH_3), 4.03 (q, *J* = 7.2 Hz, 2H, OCH_2), 7.39-7.58 (m, 5H, Ph), 8.41 (d, *J* = 13.8 Hz, 1H, H-3), 10.67 (d, *J* = 13.8 Hz, 1H, NH); ^{13}C NMR (126 MHz, DMSO-d_6) δ *E*: 13.78 (CH_3), 59.37 (OCH_2), 101.28 (C-2), 127.16 (C*o*), 127.58 (C*m*), 130.31 (C*p*), 141.49 (C*i*), 152.18 (C-3), 159.25 (C-5'), 166.59 (COO), 195.22(CO), *Z*: 13.63 (CH_3), 59.36 (OCH_2),

101.76 (C-2), 127.86(C*o*), 128.07 (C*m*), 130.99 (C*p*), 140.74 (C*i*), 151.95 (C-3), 159.44 (C-5'), 166.87 (COO), 192.37 (CO); MS (EI) m/z (%): 269 ($[M-H_2O]^+$, 7), 241 (1), 213 (2), 196 (2), 129 (6), 105 (62), 77 (24), 45 (100); Anal calcd for $C_{13}H_{13}N_5O_3$: C, 54.35; H, 4.56; N, 24.38; found: C, 54.30; H, 4.55; N, 24.40.

Ethyl 7-(1-ethoxy-1,3-dioxo-3-phenylpropan-2-yl)-5-phenyl-4,7-dihydrotetrazolo [1,5-*a*]pyrimidine-6-carboxylate (10): White crystals; yield 0.18 g (26%); mp 109-111 °C; DRIFT (cm^{-1}) ν 3202, 3151 (O–H), 3073, 3034, 2980 (C–H), 1747, 1674 (C=O), 1637, 1568 (C=C); ^1H NMR (500 MHz, $CDCl_3$) δ 0.88 (t, *J* = 7.2 Hz, 3H, CH_3), 1.15 (t, *J* = 7.2 Hz, 3H, CH_3'), 3.97 (dq, *J* = 10.7, 7.2 Hz, 1H, OCH^B), 4.01 (dq, *J* = 10.7, 7.2 Hz, 1H, OCH^A), 4.11 (q, *J* = 7.2 Hz, 2H, OCH_2'), 5.28 (d, *J* = 2.8 Hz, 1H, H-2'), 6.47 (d, *J* = 2.8 Hz, 1H, H-7), 7.39 (m, 2H, H*o*), 7.45–7.53 (m, 3H, H*m* and H*p*), 7.55 (m, 2H, H*m*'), 7.63 (m, 1H, H*p*'), 8.18 (m, 2H, H*o*'), 10.48 (s, 1H, NH); ^{13}C NMR (126 MHz, $CDCl_3$) δ 13.42 (CH_3'), 13.89 (CH_3), 55.74 (C-7), 59.03 (C-2'), 60.79 (OCH_2'), 61.69 (OCH_2), 95.60 (C-6), 128.05 (C*o*), 128.55 (C*m*), 128.98 (C*o*' and C*m*'), 130.40 (C*p*), 133.73 (C*p*'), 134.53 (C*i*), 135.62 (C*i*'), 149.74 (C-3a), 150.39 (C-5), 165.66 (COO), 167.53 (C-1'), 192.91 (C-3'); MS (EI) m/z: 269 ($[M-192]^+$, 10), 241 (1), 213 (2), 192 ($[PhOCCH_2CO_2Et]+$, 8), 129 (4), 105 (100), 77 (30); Anal calcd for $C_{24}H_{23}N_5O_5$: C, 62.46; H, 5.02; N, 15.18; found: C, 62.18; H, 5.00; N, 15.11.

5-[2,6-Diamino-3,5-bis(ethoxycarbonyl)pyridinium-1-yl]tetrazol-1-ide (11):

Method A. A mixture of ethyl 2-ethoxymethylenecyanoacetate (**1f**) (0.51 g, 3 mmol), 5-aminotetrazole (0.31 g, 3 mmol) and triethylamine (0.5 ml) in ethanol (or trifluoroethanol) (25 ml) was refluxed for 40 min. After the completion of the reaction (TLC monitoring), the reaction mixture was evaporated to dryness under reduced pressure; the resulting precipitate was filtered off and crystallized from acetonitrile to give 0.42 g (44%) (in EtOH), 0.40 g (42%) (in TFE) compound **11.**

Method B. A mixture of ethyl 2-ethoxymethylenecyanoacetate (**1f**) (0.17 g, 1 mmol), ethyl cyanoacetate (**12**) (0.11 g, 1 mmol), 5-aminotetrazole (0.09 g, 1 mmol) and triethylamine (0.2 ml) in ethanol (20 ml) was refluxed for 50 min. After the completion of the reaction (TLC monitoring), the reaction mixture was evaporated to dryness under reduced pressure; the resulting precipitate was filtered off and crystallized from acetonitrile to afford compound **11** as white crystals, mp 228–230 °C; yield 0.29 g (90%); DRIFT (cm^{-1}) ν 3292, 3238 (NH_2), 1699 (C=O), 1649–1587 (C=C, C=N); ^1H NMR (500 MHz, DMSO-d_6) δ 1.33 (t, J = 7.1 Hz, 6H, CH_3), 4.34 (q, J = 7.1 Hz, 4H, OCH_2), 8.24 (s, 2H, NH), 8.77 (s, 1H, H-4), 9.33 (s, 2H, NH); ^{13}C NMR (126 MHz, DMSO-d_6) δ 14.03 (CH_3), 61.61 (OCH_2), 95.49 (C-3, 5), 146.41 (C-4), 151.82 (C-5'), 154.92 (C-2, 6), 164.51 (COO); MS (EI) m/z (%): 321 (M+, 31), 276 (12), 265 (37), 236 (54), 208 (31), 190 (100), 146 (92), 120 (52), 107 (20), 91 (32), 79 (21), 64 (37), 52 (43); Anal calcd for $C_{12}H_{15}N_7O_4$: C, 44.86; H, 4.71; N, 30.52; found: C, 44.75; H, 4.61; N, 30.60.

Diethyl 2,4-dicyanopent-2-enedioate monohydrate (14). A mixture of ethyl 2-ethoxymethylenecyanoacetate (**1f**) (0.17 g, 1 mmol), ethyl cyanoacetate (**12)** (0.11 g, 1 mmol) and triethylamine (0.2 ml) in ethanol (15 ml) was refluxed for 60 min. After the completion of the reaction (TLC monitoring), the reaction mixture was evaporated, the resulting precipitate was washed with chloroform and dried to give 0.21 g (89%) of compound **14** as a white powder; mp 260–262 °C; DRIFT (cm^{-1}) ν 3502 (H_2O), 2982, 2934 (C-H), 2213, 2189 (C≡N), 1678, 1662 (C=O), 1532 (C=C); ^1H NMR (500 MHz, DMSO-d_6) δ 1.17 (t, J = 7.1 Hz, 6H, 2 CH_3), 4.05 (q, J = 7.1 Hz, 4H, 2 OCH_2), 8.01 (s, 1H, CH); ^{13}C NMR (126 MHz, DMSO-d_6) δ 14.50 (CH_3), 59.07 (OCH_2), 72.55 (C-3), 117.93 (C-2, C-4), 150.05 (CN), 166.53 (C-1, C-5); MS (EI) m/z (%): 236 (M^+, 76), 221 (36), 193 (81), 165 (46), 147 (41), 138 (72), 120 (20), 109 (19), 95 (28), 68 (54), 44

(100); Anal calcd for $C_6H_{11}N_5O_3$: C, 51.97; H, 5.55; N, 11.02; found: C, 51.45; H, 5.58; N, 10.96.

Crystallographic details

The single crystals of the compounds **5** and **11** were obtained by crystallization from acetone-ethanol 2:1 and acetonitrile, respectively. The X-ray studies were performed on an Xcalibur 3 CCD diffractometer at 295(2) K (MoKα irradiation, graphite monochromator, CCD detector, $\omega/2\theta$ scanning). The crystal structures were solved by direct methods followed by Fourier synthesis with SHELXS-97 and refined with full-matrix least squares methods for all non-hydrogen atoms with SHELXL-97 software packages [5].

Crystallographic data for 5: $C_9H_8F_3N_7O_2$ (M = 393.23) are monoclinic, space group $P2(1)/c$, a = 13.6445(10) Å, b = 8.2009(16) Å, c = 12.3719(17) Å, $\alpha = \gamma = 90$ °, β = 116.625(11)°, V = 1237.6(3) Å^3, Z = 4, Dcalc = 1.627 g/cm^3, μ(MoKα) = 0.133 mm^{-1}, F(000) = 616, 8453 reflections measured, 2256 unique reflections which were used in all calculations. The final R is 0.0596, number of refined parameters 227.

Crystallographic data for 11: $C_{12}H_{15}N_7O_4$ H_2O C_2H_3N (M = 380.38) are monoclinic, space group $P2(1)/c$, a = 12.4718(7) Å, b = 14.4203(11) Å, c = 9.7541(9) Å, $\alpha = \gamma = 90°$, β = 100.554(7)°, V = 1724.6(2) Å^3, Z = 4, Dcalc = 1.465 g/cm^3, μ(MoKα) = 0.114 mm^{-1}, F(000) = 800, 11671 reflections measured, 2184 unique reflections which were used in all calculations. The final R is 0.0541, number of refined parameters 278.

CCDC Nos. 918900 and 1022736 contain the supplementary crystallographic data for **5** and **11**, respectively. These data can be obtained free of charge via http://www.ccdc.cam.ac.uk/conts/retrieving.html (or from the CCDC, 12 Union Road, Cambridge CB2 1EZ, UK; fax: +44 1223 336033; e-mail: deposit@ccdc.cam.ac.uk).

References

1. Pryadeina, M. V.; Burgart, Ya. V.; Saloutin, V. I.; Slepukhin, P. A.; Kazheva, O. N.; Shilov, G. V.; D'yachenko, O. A.; Chupakhin, O. N. *Russ. J. Org. Chem*. **2007**, *43*, 945–955. doi: 10.1134/S1070428007070019
2. Ackermann, O.; Bretzinger, D.; Schneidewind, H.; Stephan R. Alkoxymethylene compounds. US Patent 4277418 A, July 7, 1981; *Chem. Abstr.* **1981**, *95*, 149983.
3. Misra, R. N.; Rawlins, D. B.; Xiao, H.-y.; Shan, W.; Bursuker, I.; Kellar, K. A.; Mulheron, J. G.; Sack, J. S.; Tokarski, J. S.; Kimball, S. D.; Webster. K. R. *Bioorg. Med. Chem. Lett.* **2003**, *13*, 1133–1136. doi: 10.1016/S0960-894X(03)00034-9
4. Sansebastiano, L.; Mosti, L; Menozzi, G.; Schenone, P.; Muratore, O.; Petta, A.; Debbia, E.; Schito, A. P.; Schito, G. C. *Farmaco, Ed. Sci.* **1993**, *48*, 335–355.
5. Sheldrick, G. M. Acta Crystallogr., Sect. A 2008, A64, 112–122. doi: 10.1107/S0108767307043930

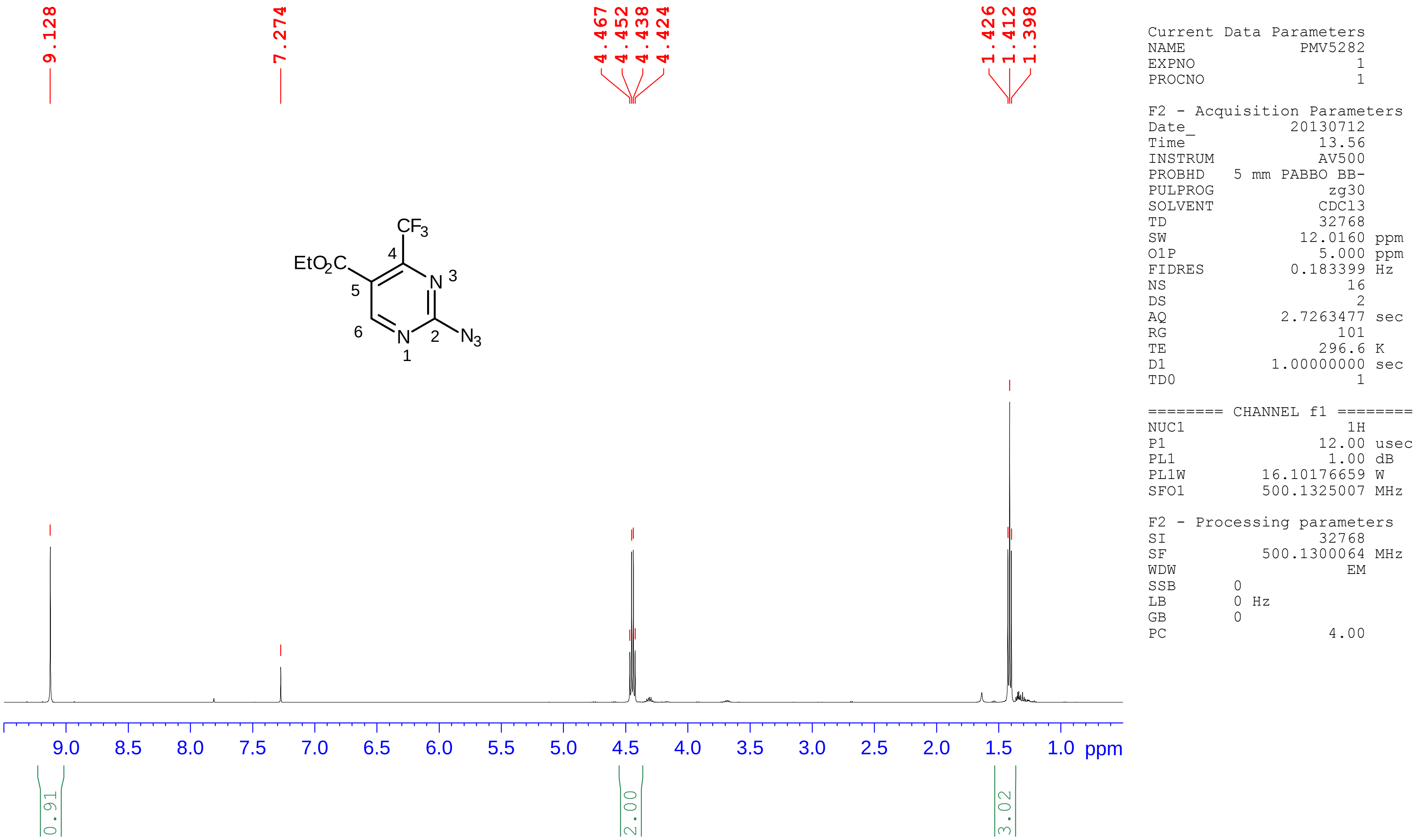

^{1}H NMR (500 MHz, $CDCl_3$) spectrum of **2a.**

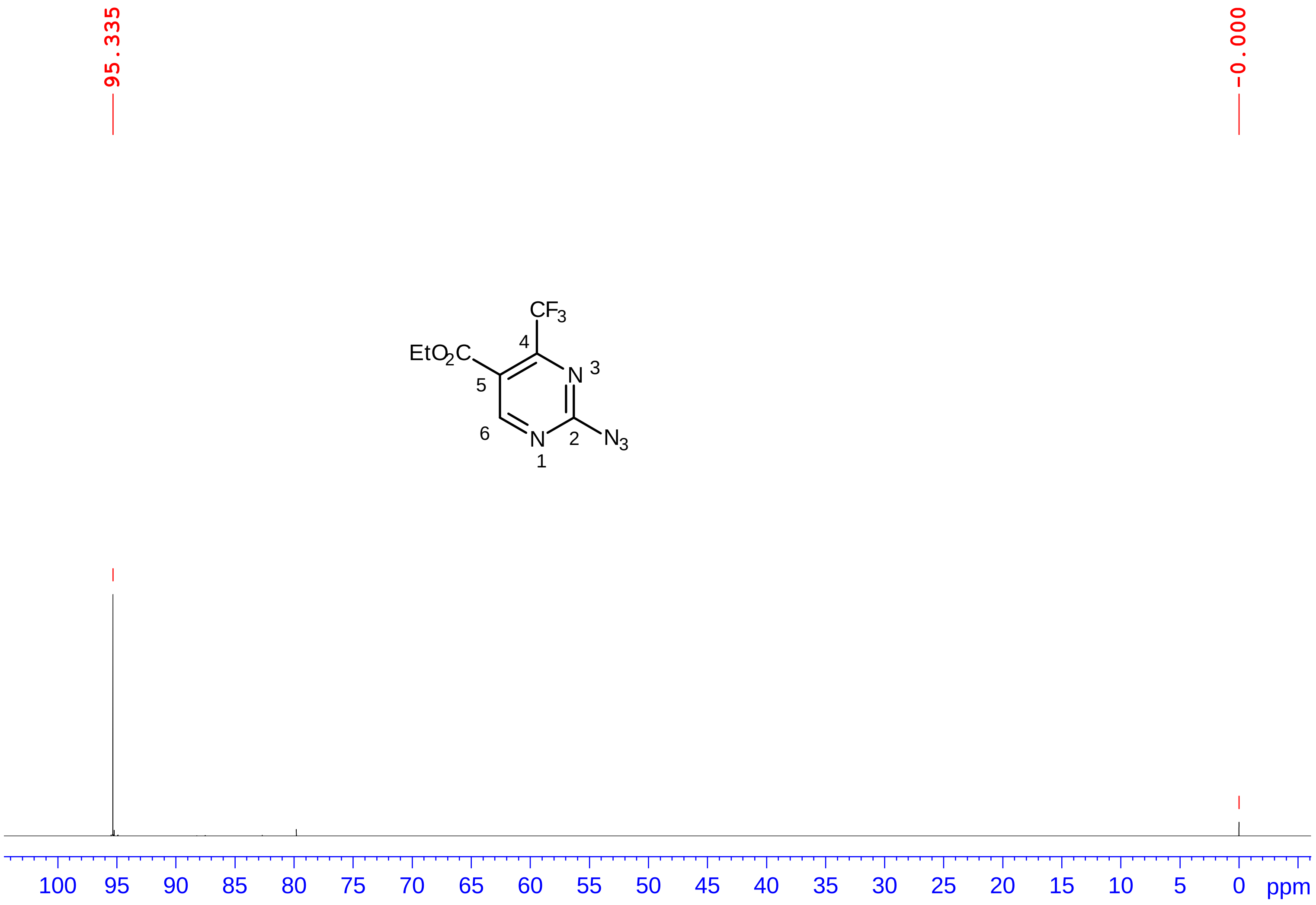


```
Current Data Parameters
NAME               PMV5282
EXPNO                   19
PROCNO                   1

F2 - Acquisition Parameters
Date_             20130712
Time                 14.06
INSTRUM              AV500
PROBHD   5 mm PABBO BB-
PULPROG             zgflqn
SOLVENT              CDCl3
TD                  262144
SW                110.6886 ppm
O1P                 50.000 ppm
FIDRES            0.198682 Hz
NS                      16
DS                       2
AQ               2.5166323 sec
RG                     203
TE                   296.6 K
D1              1.00000000 sec

======== CHANNEL f1 ========
NUC1                   19F
P1                    5.00 usec
PL1                  -5.00 dB
PL1W           46.07103729 W
SFO1           470.5394058 MHz

F2 - Processing parameters
SI                  262144
SF             470.5162425 MHz
WDW                     EM
SSB      0
LB                    0.25 Hz
GB       0
PC                    1.50
```

^{19}F NMR (470 MHz, $CDCl_3$) spectrum of **2a.**

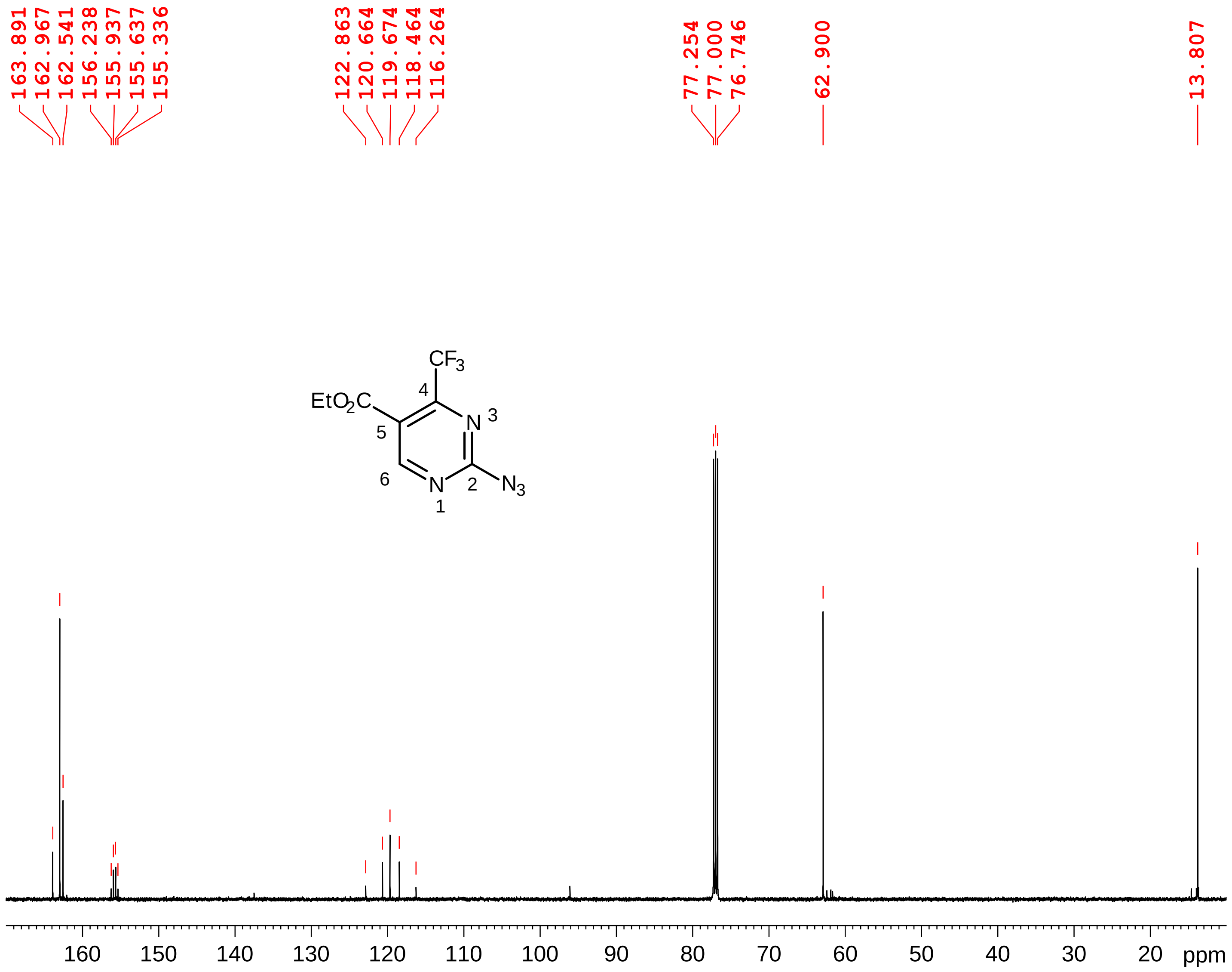


```
Current Data Parameters
NAME                PMV5282
EXPNO                    13
PROCNO                    1

F2 - Acquisition Parameters
Date_              20130712
Time                  14.14
INSTRUM               AV500
PROBHD     5 mm PABBO BB-
PULPROG             zgpg30
SOLVENT               CDCl3
TD                    65536
SW                 200.7838 ppm
O1P                  95.000 ppm
FIDRES             0.385323 Hz
NS                     1024
DS                        8
AQ                1.2976629 sec
RG                      203
TE                    297.1 K
D1               0.80000001 sec
D11              0.03000000 sec
TD0                       1

======== CHANNEL f1 ========
NUC1                    13C
P1                     9.00 usec
PL1         0 dB
PL1W        115.29558563 W
SFO1         125.7697360 MHz

======== CHANNEL f2 ========
CPDPRG2             waltz16
NUC2                     1H
PCPD2                 75.00 usec
PL2                  120.00 dB
PL12                  17.00 dB
PL13                  20.00 dB
PL2W        0 W
PL12W            0.40445811 W
PL13W            0.20270923 W
SFO2           500.1320005 MHz

F2 - Processing parameters
SI                    65536
SF             125.7577915 MHz
WDW                      EM
SSB         0
LB                     1.00 Hz
GB          0
PC                     1.40
```

^{13}C NMR (126 MHz, $CDCl_3$) spectrum of **2a.**

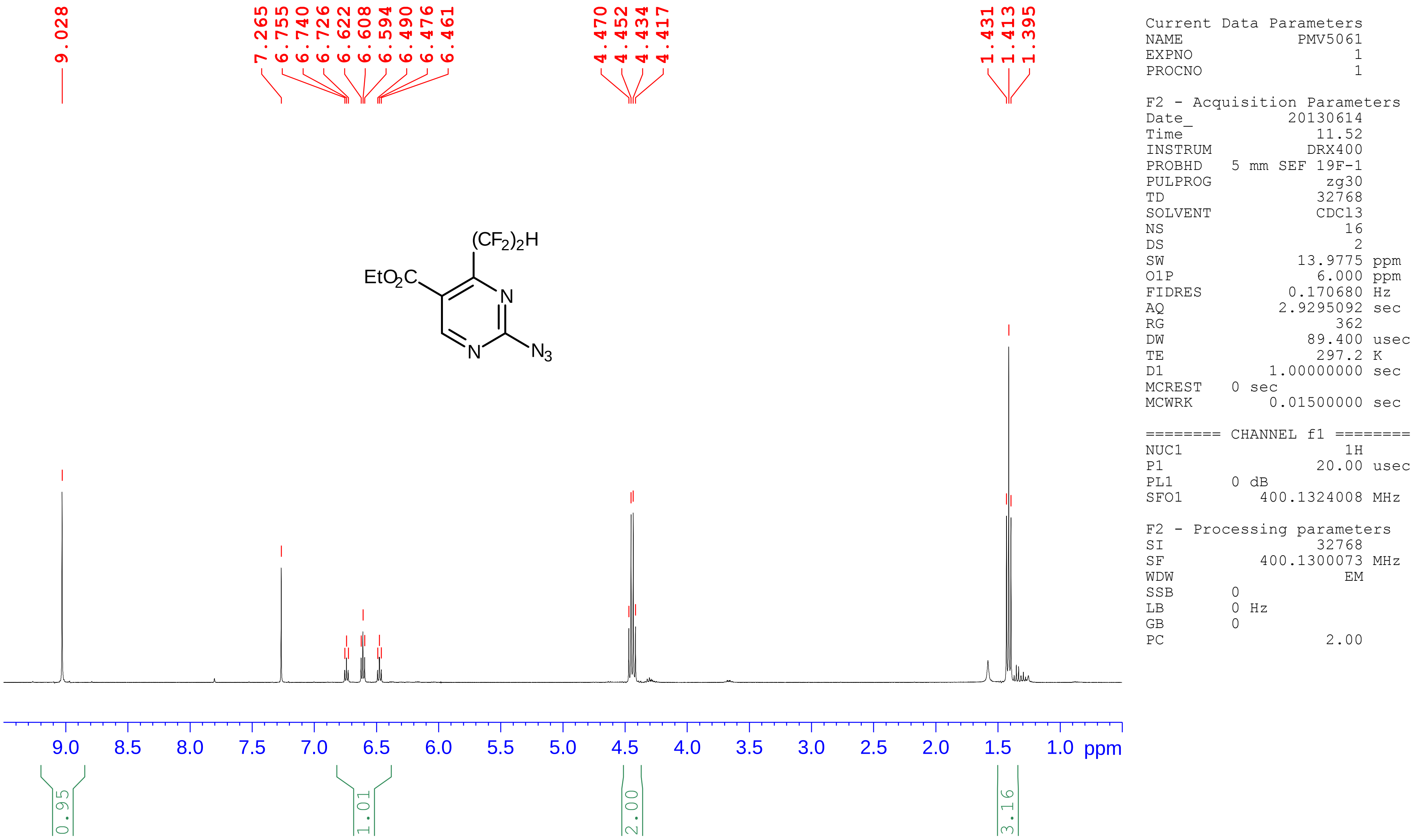

^{1}H NMR (400 MHz, $CDCl_3$) spectrum of **2b.**

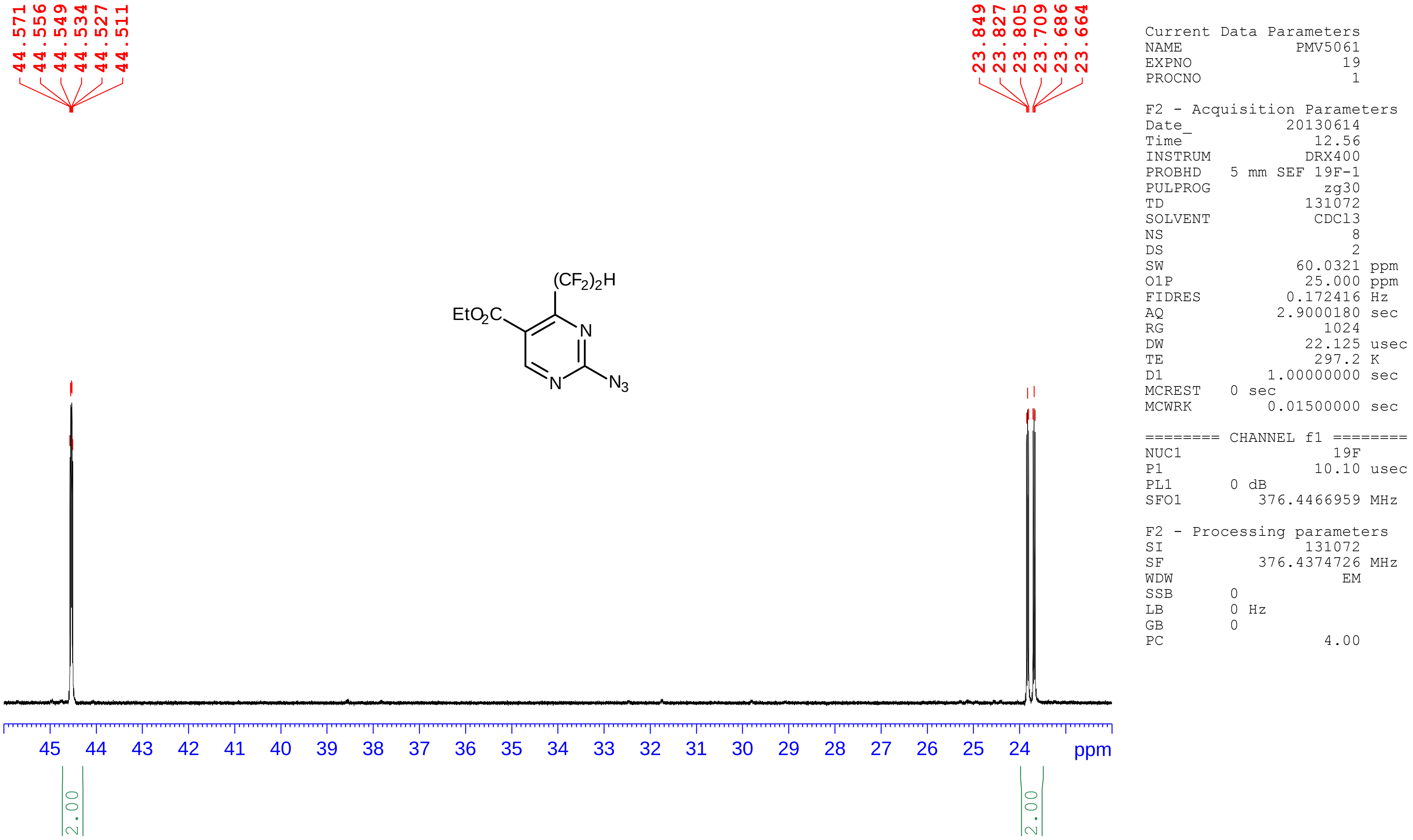

^{19}F NMR (376 MHz, $CDCl_3$) spectrum of **2b.**

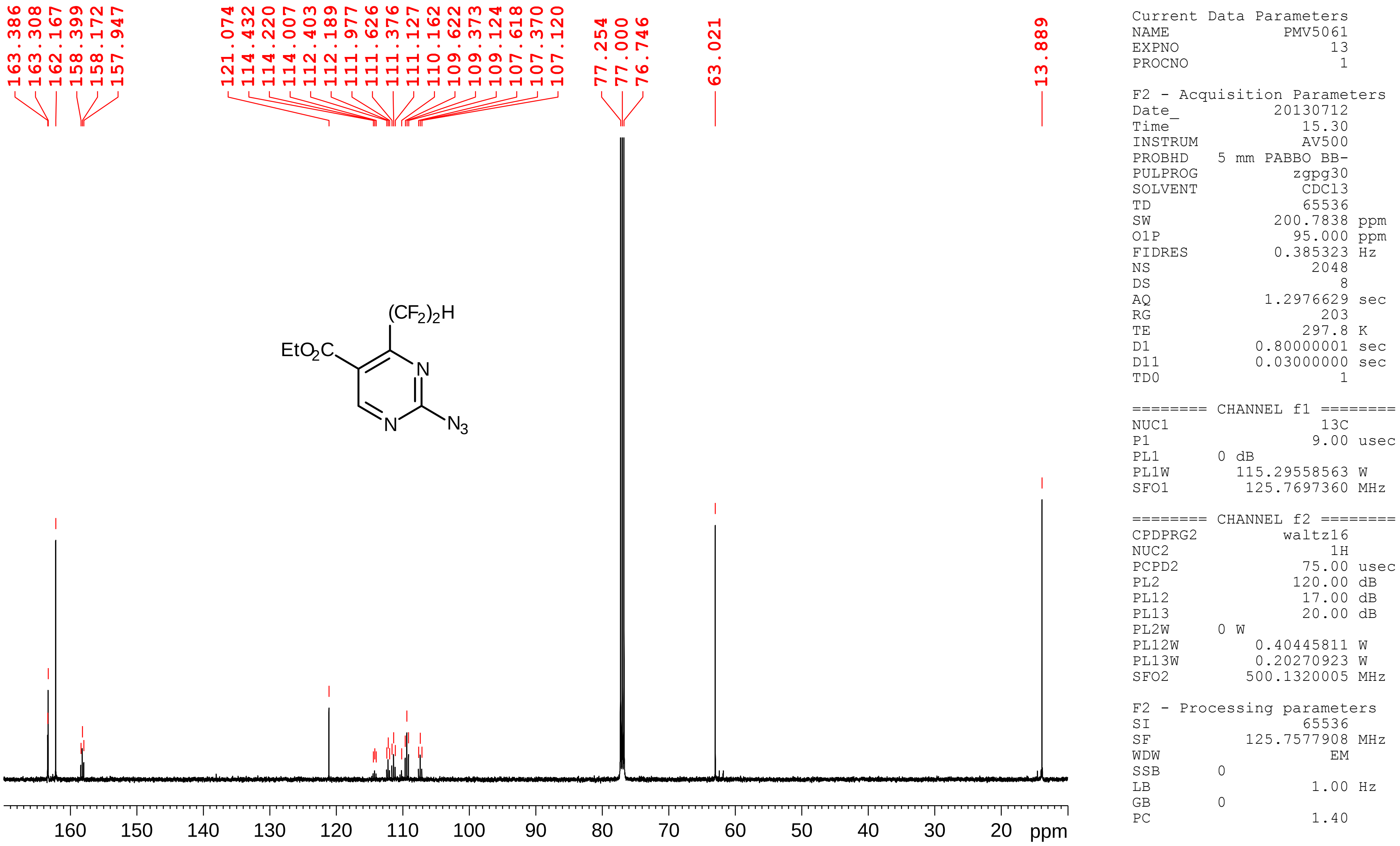

^{13}C NMR (126 MHz, $CDCl_3$) spectrum of **2b.**

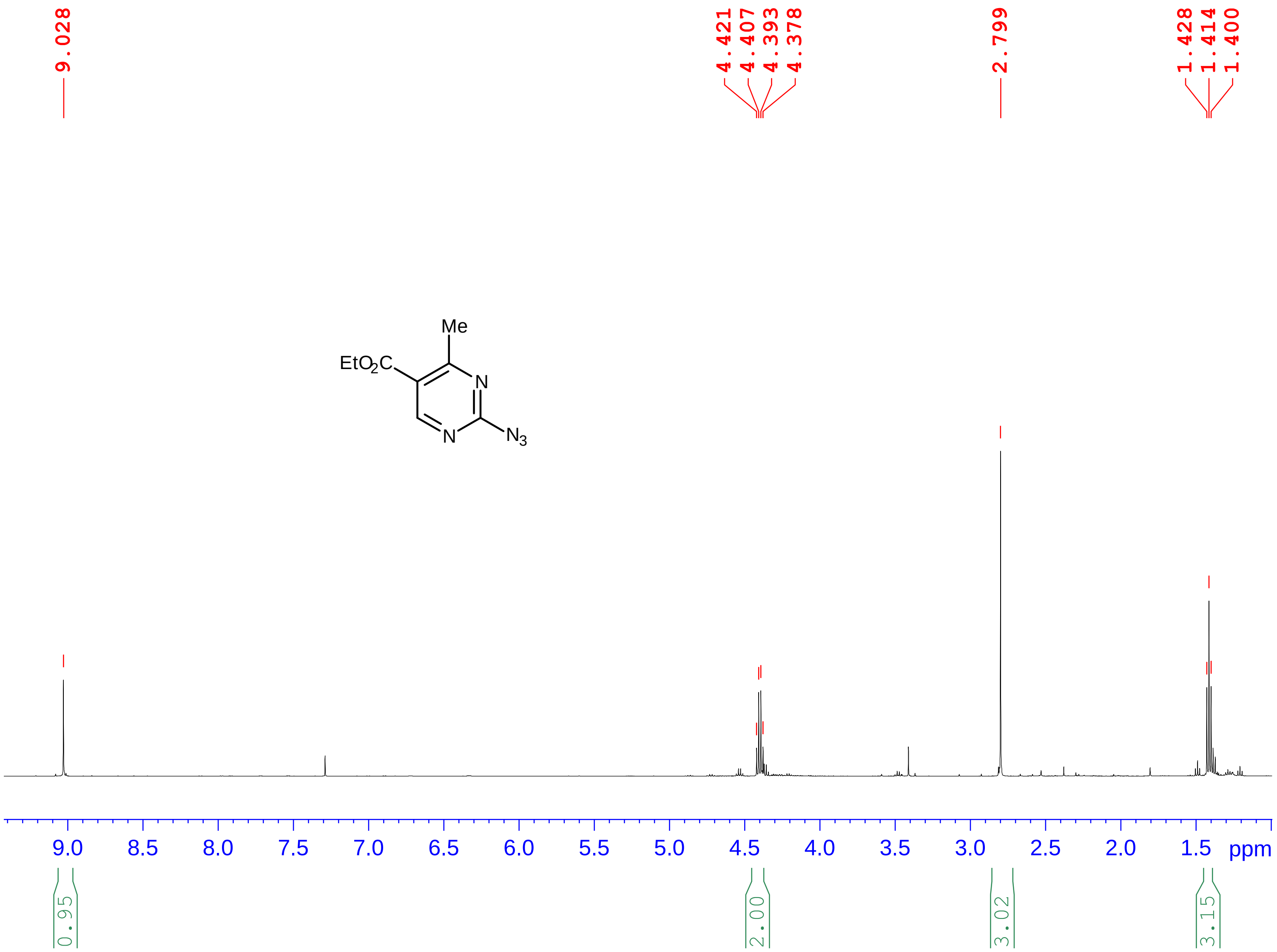


```
Current Data Parameters
NAME              PMV52912
EXPNO                    1
PROCNO                   1

F2 - Acquisition Parameters
Date_             20140402
Time                 15.52
INSTRUM              AV500
PROBHD   5 mm PABBO BB-
PULPROG               zg30
SOLVENT              CDCl3
TD                   65536
SW                 19.9946 ppm
O1P                  9.000 ppm
FIDRES            0.152588 Hz
NS                      16
DS                       2
AQ               3.2768500 sec
RG                      64
TE                   296.7 K
D1              1.00000000 sec
TD0                      1

======== CHANNEL f1 ========
NUC1                    1H
P1                   12.00 usec
PL1                   1.00 dB
PL1W           16.10176659 W
SFO1           500.1345012 MHz

F2 - Processing parameters
SI                   32768
SF             500.1299996 MHz
WDW                     EM
SSB        0
LB         0 Hz
GB         0
PC                    4.00
```

^{1}H NMR (500 MHz, $CDCl_3$) spectrum of **2c.**

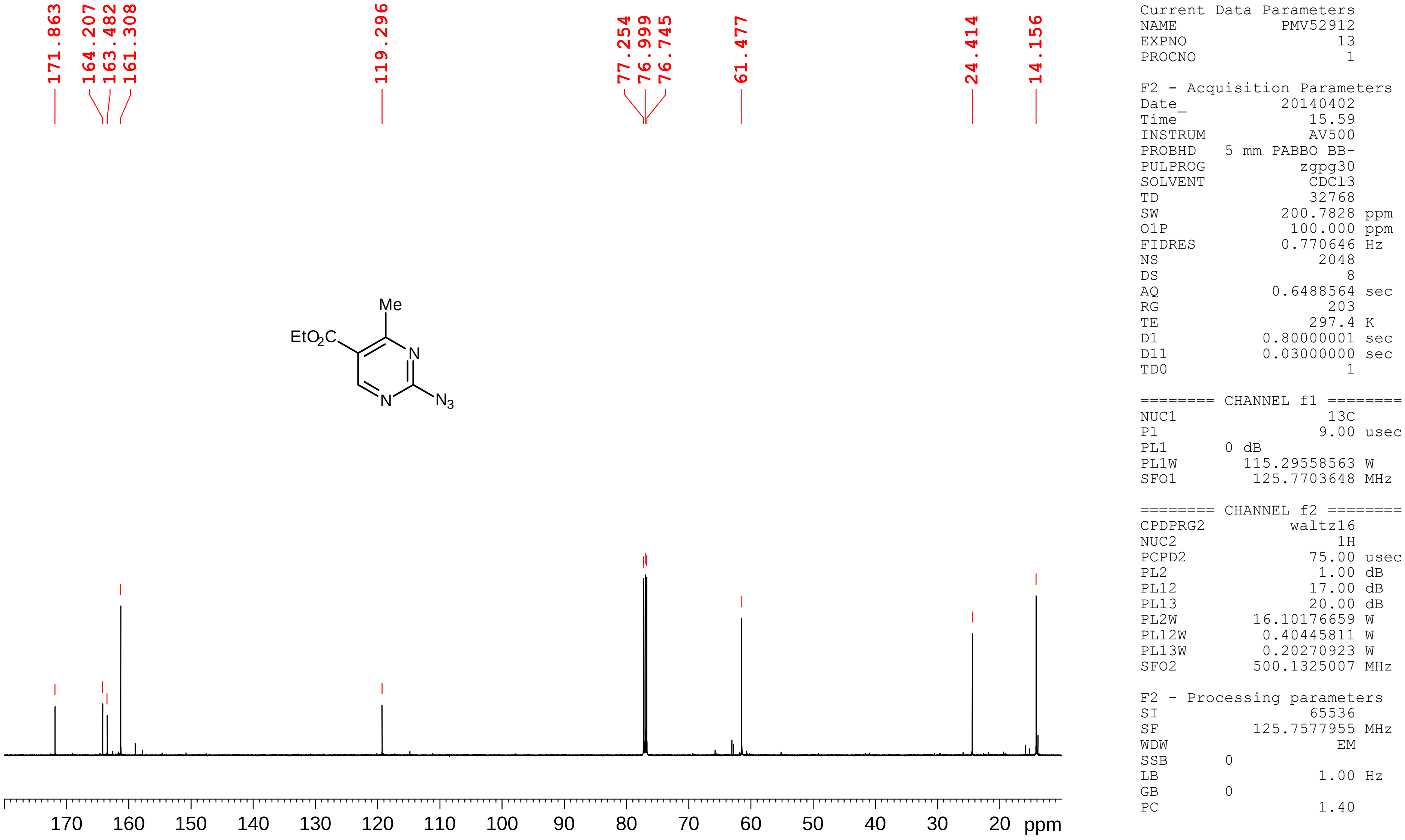

^{13}C NMR (126 MHz, $CDCl_3$) spectrum of **2c.**

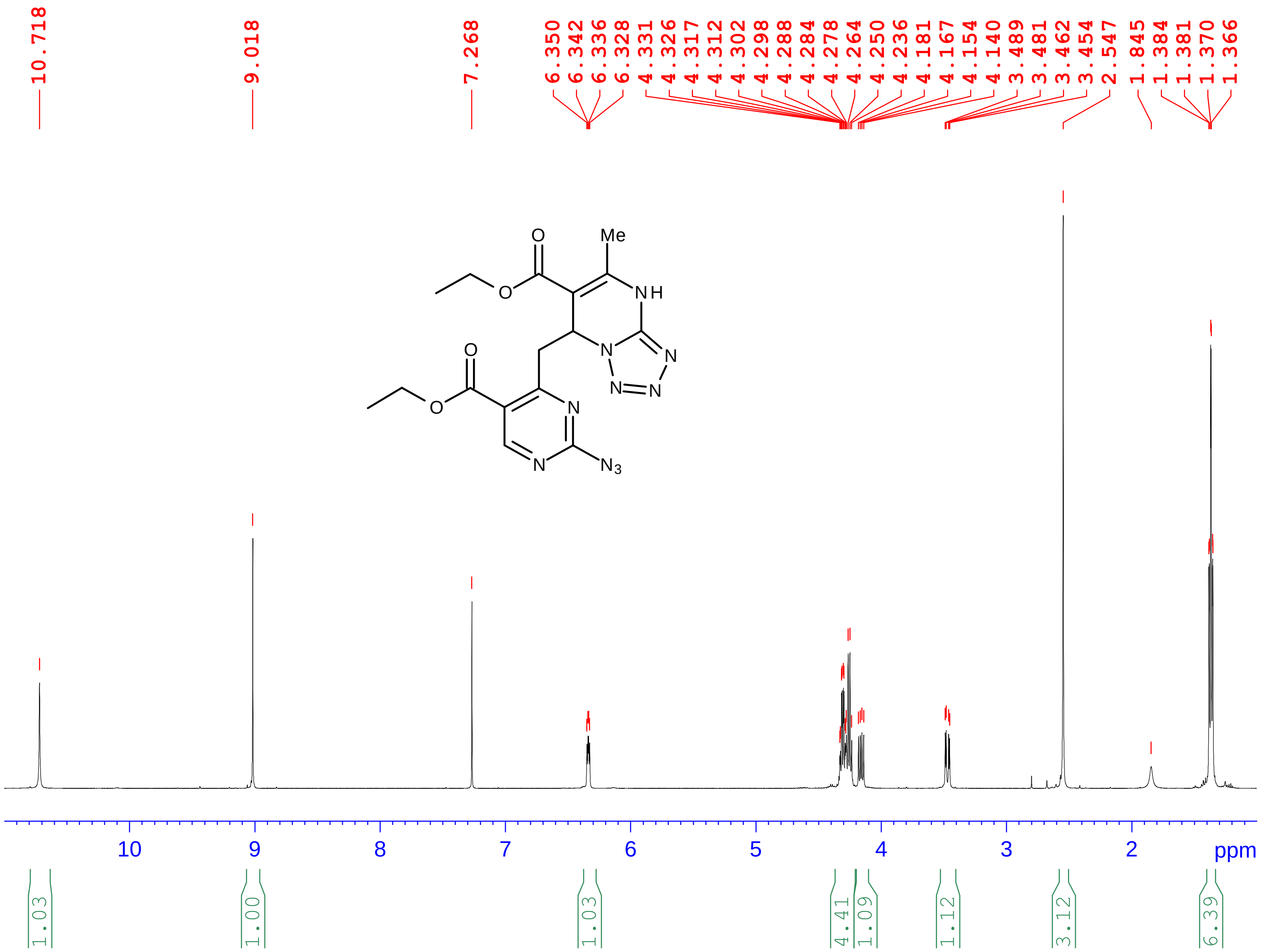

Current Data Parameters
NAME PMV5296
EXPNO 1
PROCNO 1

F2 - Acquisition Parameters
Date_ 20130919
Time 12.41
INSTRUM AV500
PROBHD 5 mm PABBO BB-
PULPROG zg30
SOLVENT CDCl3
TD 32768
SW 16.0214 ppm
O1P 7.000 ppm
FIDRES 0.244532 Hz
NS 16
DS 2
AQ 2.0447731 sec
RG 128
TE 296.5 K
D1 1.00000000 sec
TD0 1

======== CHANNEL f1 ========
NUC1 1H
P1 12.00 usec
PL1 1.00 dB
PL1W 16.10176659 W
SFO1 500.1335009 MHz

F2 - Processing parameters
SI 32768
SF 500.1300096 MHz
WDW EM
SSB 0
LB 0 Hz
GB 0
PC 4.00

^{1}H NMR (500 MHz, $CDCl_3$) spectrum of **3.**

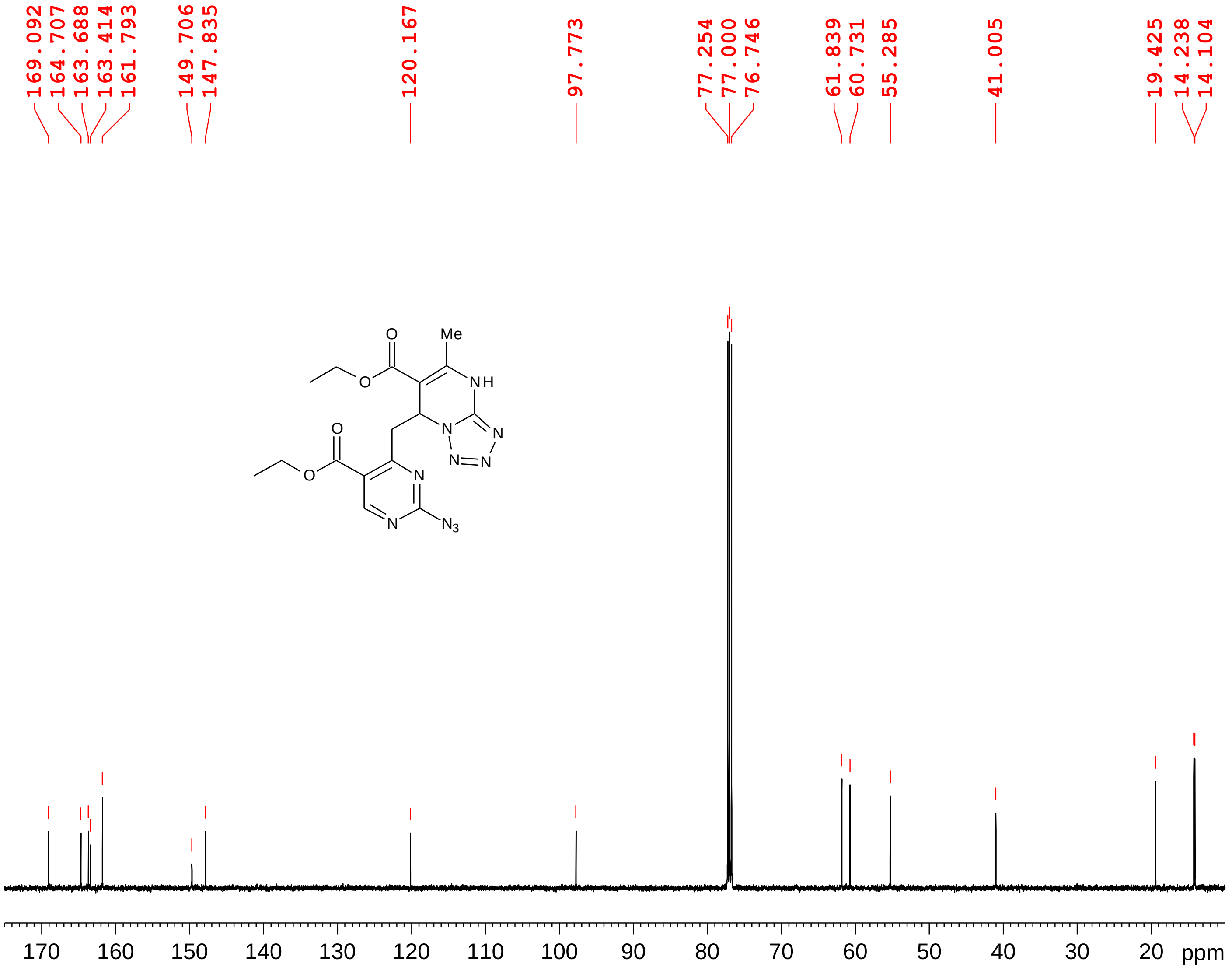


```
Current Data Parameters
NAME                PMV5296
EXPNO                    13
PROCNO                    1

F2 - Acquisition Parameters
Date_              20130920
Time                  13.08
INSTRUM               AV500
PROBHD    5 mm PABBO BB-
PULPROG             zgpg30
SOLVENT              CDCl3
TD                   32768
SW                219.2360 ppm
O1P                105.000 ppm
FIDRES            0.841477 Hz
NS                    1024
DS                       8
AQ               0.5942430 sec
RG                     203
TE                   297.9 K
D1              0.80000001 sec
D11             0.03000000 sec
TD0                      1

======== CHANNEL f1 ========
NUC1                   13C
P1                    9.00 usec
PL1       0 dB
PL1W          115.29558563 W
SFO1           125.7709936 MHz

======== CHANNEL f2 ========
CPDPRG2            waltz16
NUC2                    1H
PCPD2                75.00 usec
PL2                 120.00 dB
PL12                 17.00 dB
PL13                 20.00 dB
PL2W      0 W
PL12W           0.40445811 W
PL13W           0.20270923 W
SFO2           500.1325007 MHz

F2 - Processing parameters
SI                   65536
SF             125.7577928 MHz
WDW                     EM
SSB       0
LB                    1.00 Hz
GB        0
PC                    1.40
```

^{13}C NMR (126 MHz, $CDCl_3$) spectrum of **3.**

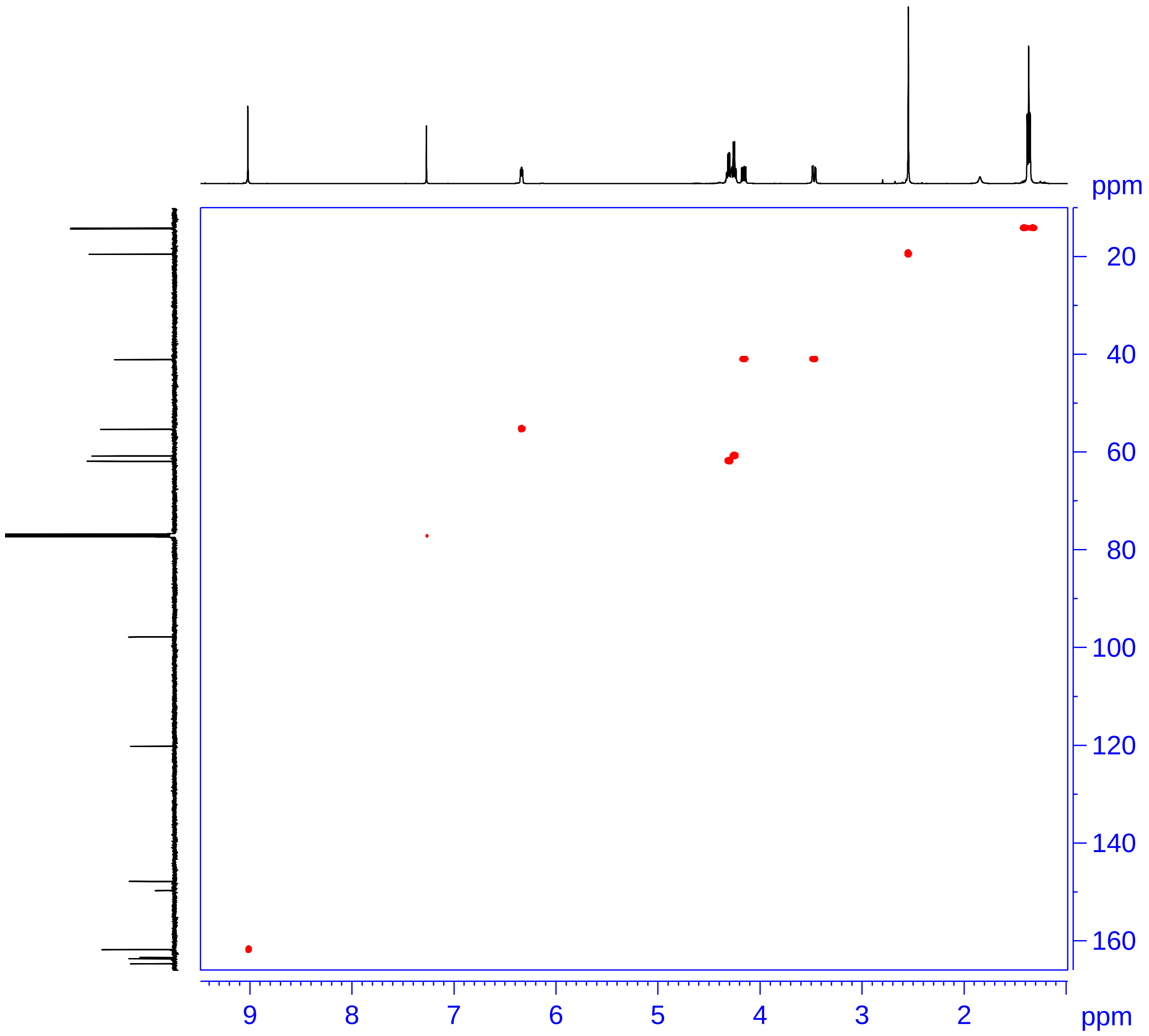

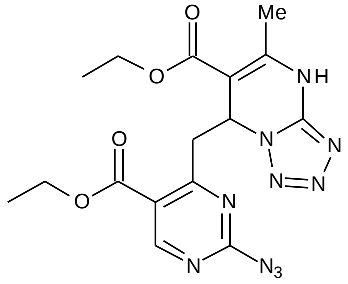

2D ^1H–^{13}C HSQC (500 MHz, $CDCl_3$) spectrum of **3**

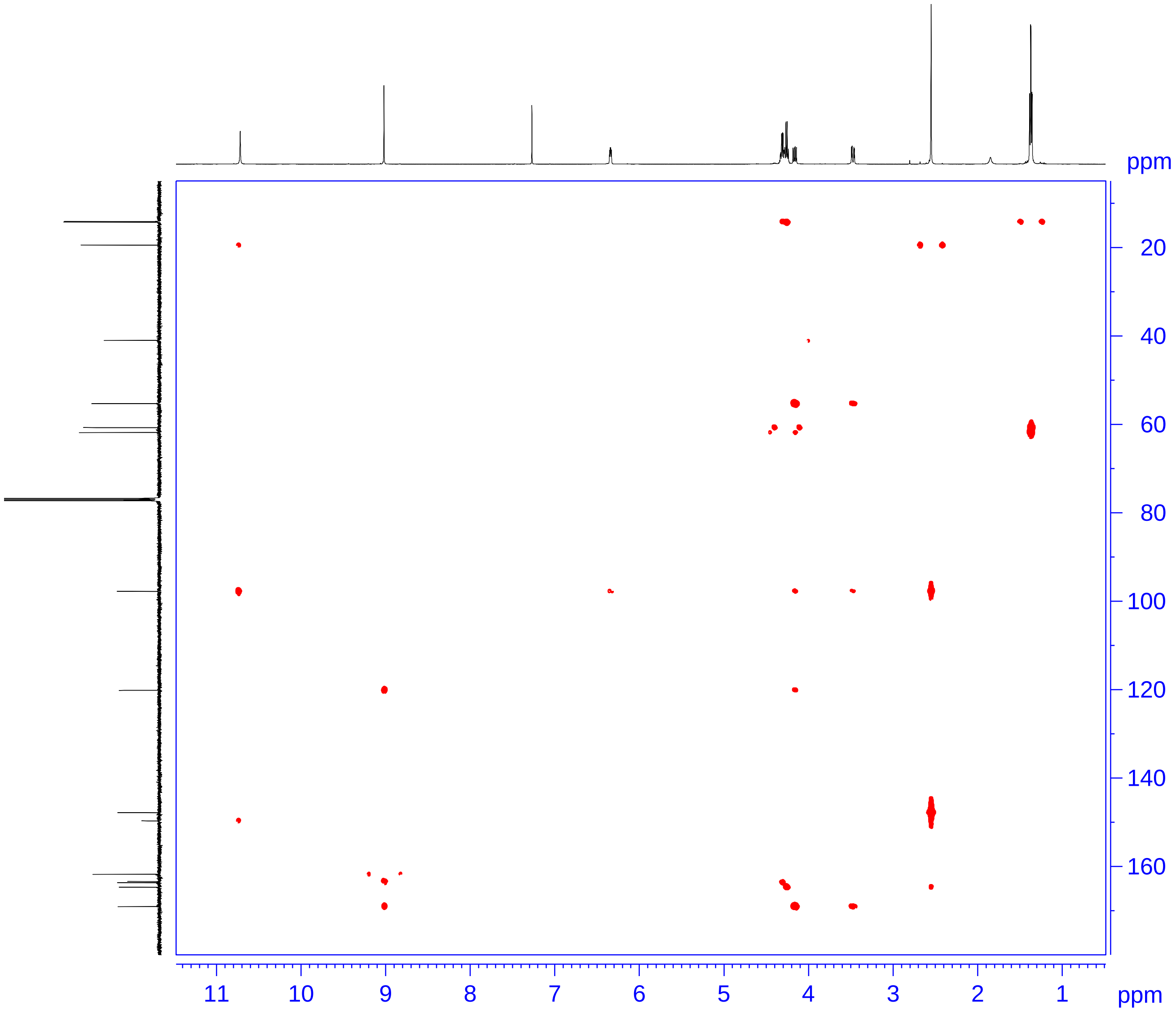

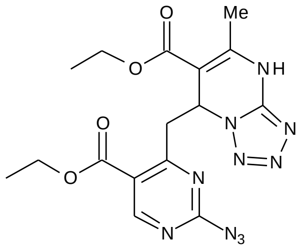

2D $^1H–^{13}C$ HMBC (500 MHz, $CDCl_3$) spectrum of **3**

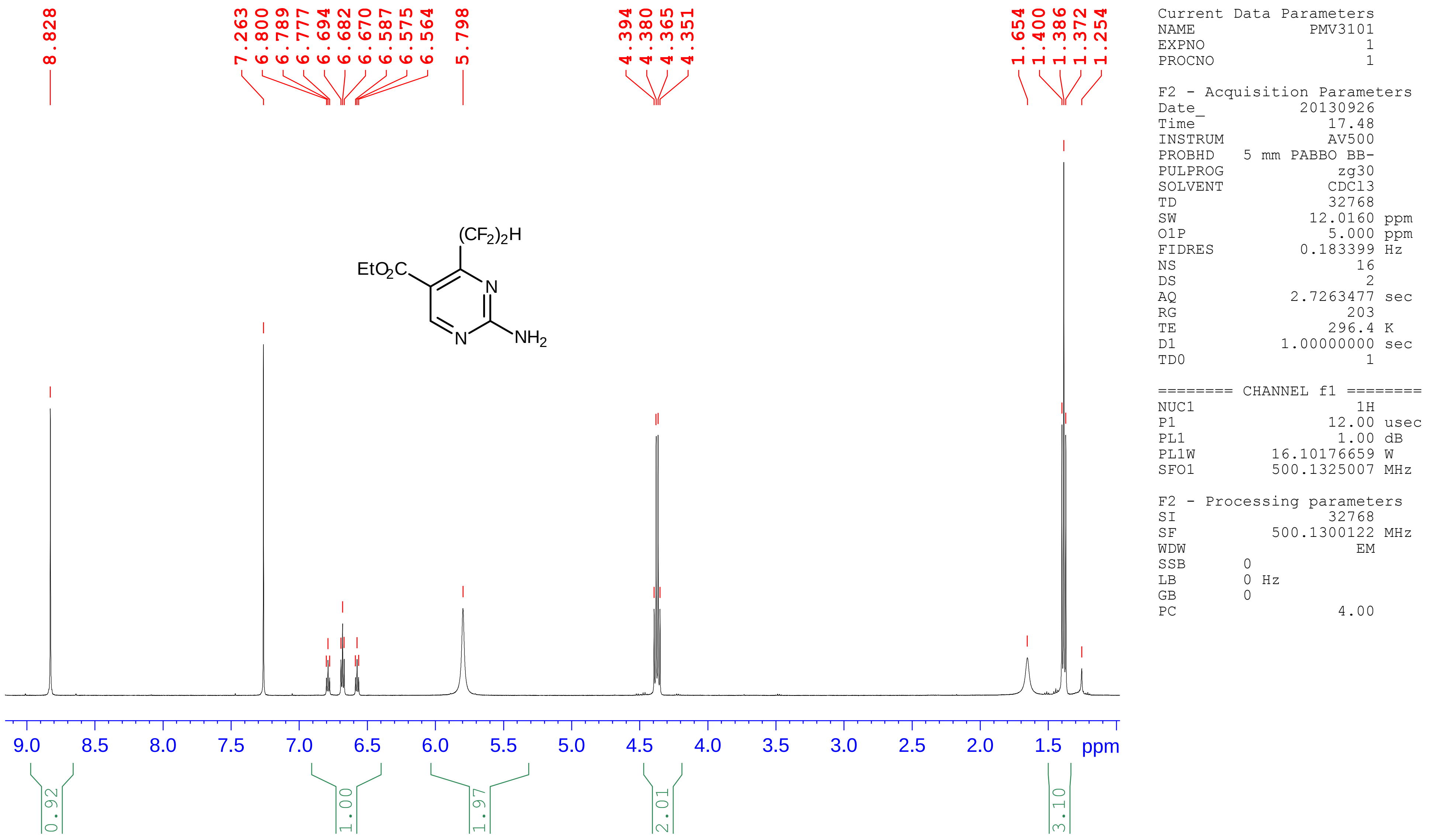

^{1}H NMR (500 MHz, $CDCl_3$) spectrum of **4b.**

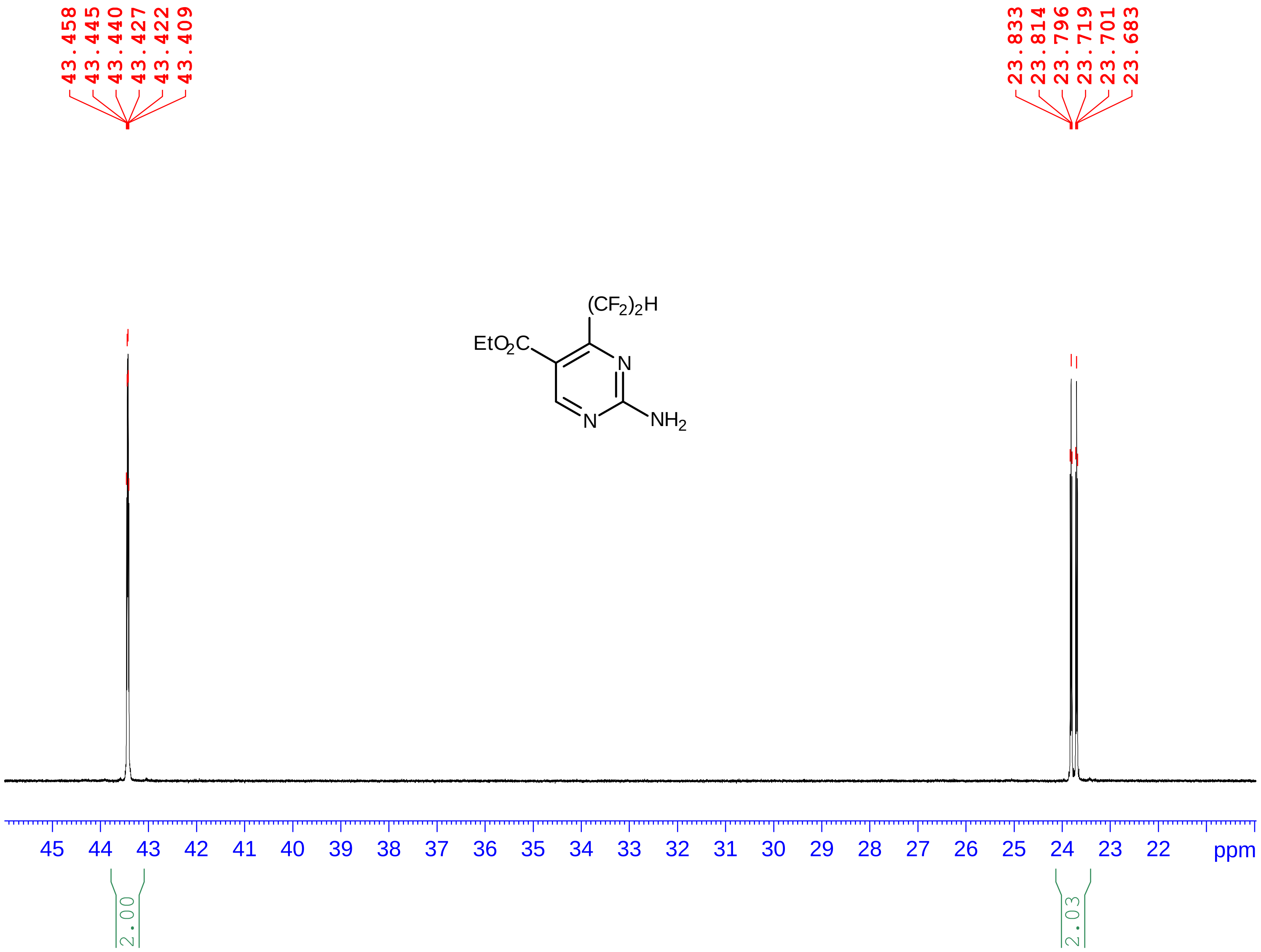


```
Current Data Parameters
NAME              PMV3101
EXPNO                  19
PROCNO                  1

F2 - Acquisition Parameters
Date_            20130926
Time                17.54
INSTRUM             AV500
PROBHD   5 mm PABBO BB-
PULPROG            zgflqn
SOLVENT             CDCl3
TD                 131072
SW                60.3771 ppm
O1P                25.000 ppm
FIDRES           0.216744 Hz
NS                     16
DS                      2
AQ              2.3069172 sec
RG                    203
TE                  296.4 K
D1             1.00000000 sec
TD0                     1

======== CHANNEL f1 ========
NUC1                  19F
P1                   5.00 usec
PL1                 -5.00 dB
PL1W          46.07103729 W
SFO1          470.5276429 MHz

F1 - Acquisition parameters
TD                    256
SFO1             125.7667 MHz
FIDRES          77.746742 Hz
SW                158.255 ppm
FnMODE        States-TPPI

F2 - Processing parameters
SI                 131072
SF            470.5162779 MHz
WDW                    EM
```

^{19}F NMR (470 MHz, $CDCl_3$) spectrum of **4b.**

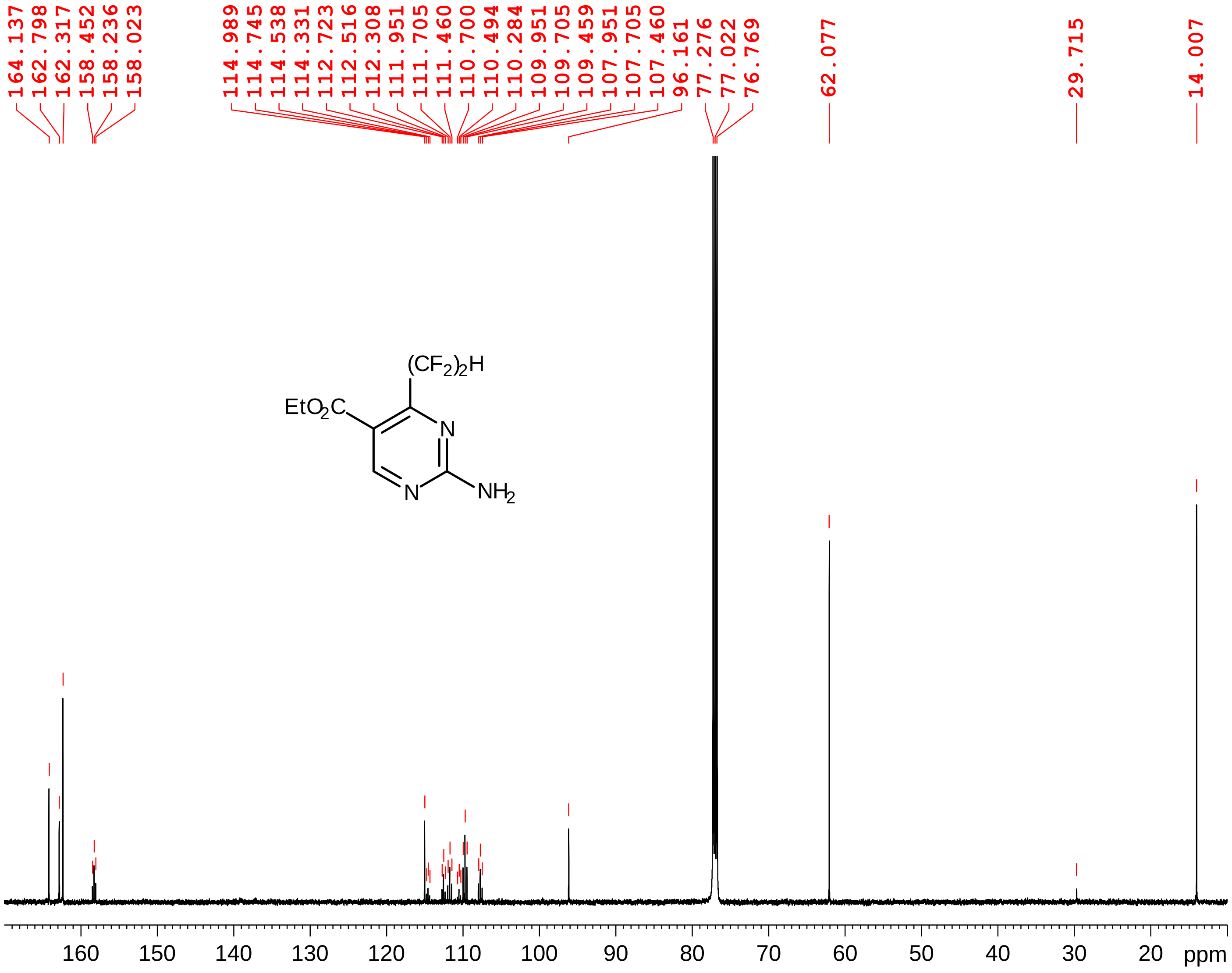


```
Current Data Parameters
NAME                 PMV3101
EXPNO                     13
PROCNO                     1

F2 - Acquisition Parameters
Date_               20130926
Time                   17.01
INSTRUM                AV500
PROBHD     5 mm PABBO BB-
PULPROG              zgpg30
SOLVENT                CDCl3
TD                     65536
SW               200.7838 ppm
O1P                95.000 ppm
FIDRES           0.385323 Hz
NS                     24576
DS                         8
AQ              1.2976629 sec
RG                       203
TE                   296.9 K
D1             0.80000001 sec
D11            0.03000000 sec
TD0                       24

======== CHANNEL f1 ========
NUC1                     13C
P1                   9.00 usec
PL1          0 dB
PL1W          115.29558563 W
SFO1          125.7697360 MHz

======== CHANNEL f2 ========
CPDPRG2              waltz16
NUC2                      1H
PCPD2               75.00 usec
PL2                120.00 dB
PL12                17.00 dB
PL13                20.00 dB
PL2W         0 W
PL12W             0.40445811 W
PL13W             0.20270923 W
SFO2          500.1320005 MHz

F2 - Processing parameters
SI                     65536
SF            125.7577853 MHz
WDW                       EM
SSB          0
LB                   1.00 Hz
GB           0
PC                      1.40
```

^{13}C NMR (126 MHz, $CDCl_3$) spectrum of **4b.**

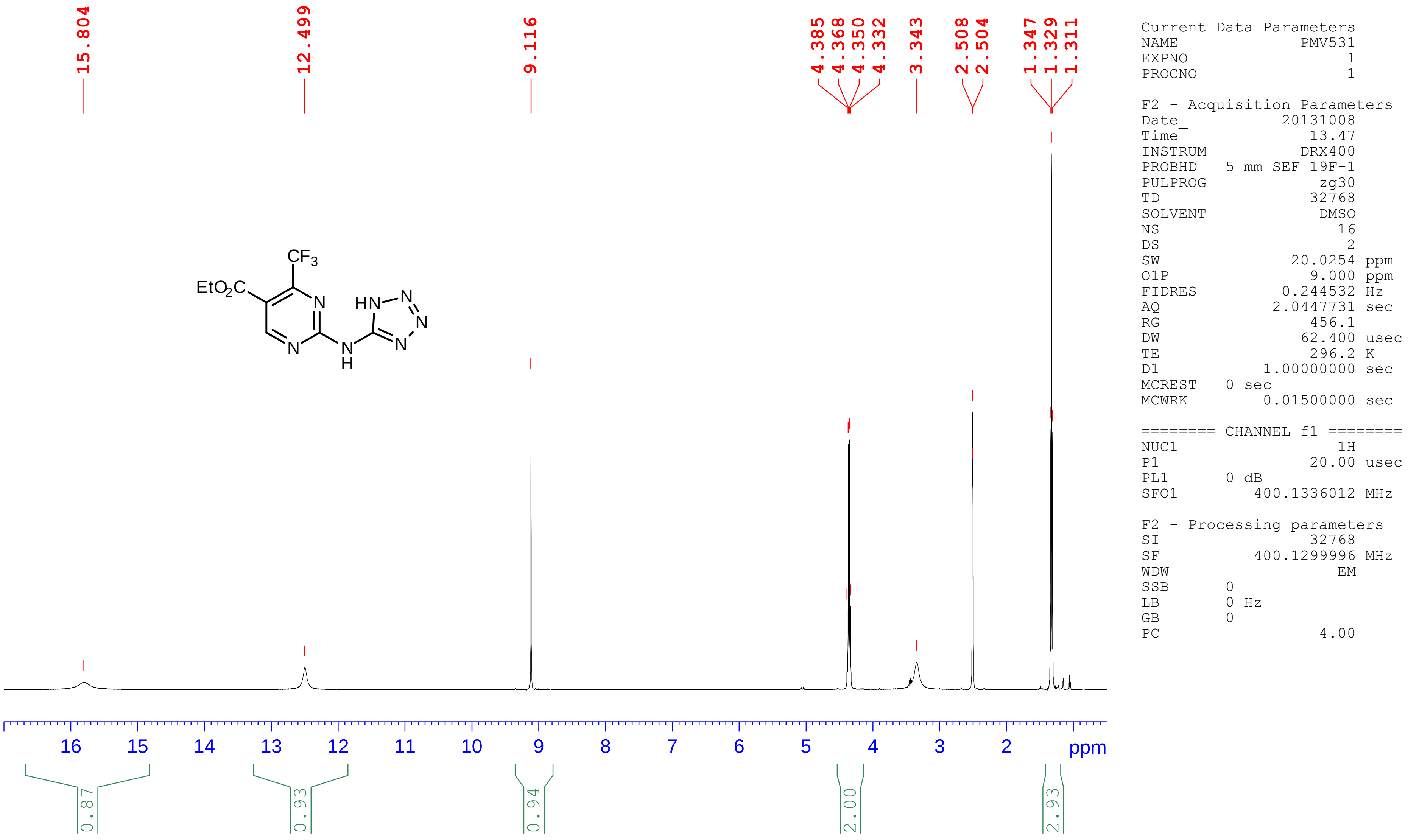

^{1}H NMR (400 MHz, DMSO-d_6) spectrum of **5.**

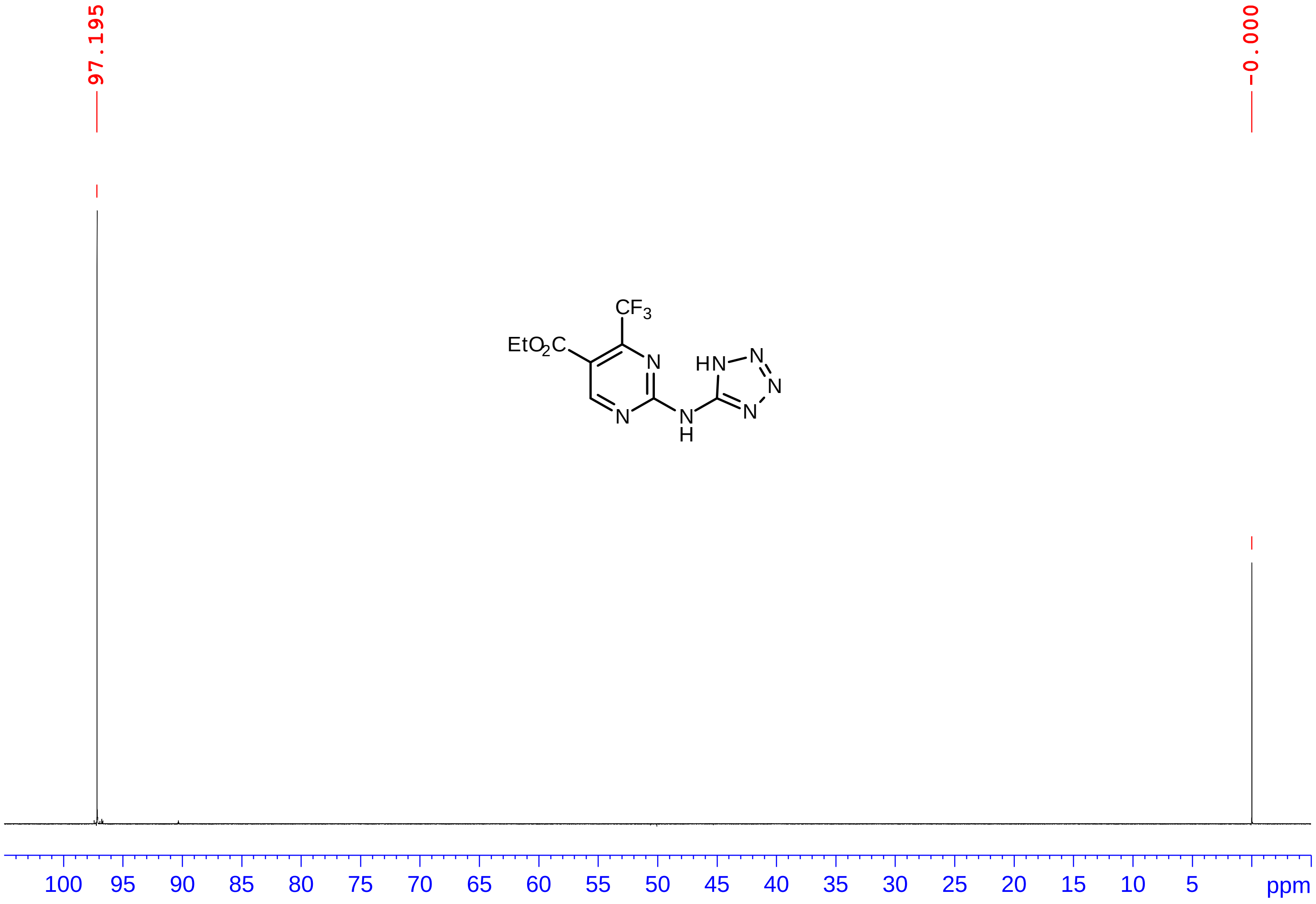


```
Current Data Parameters
NAME                 PMV531
EXPNO                    19
PROCNO                    1

F2 - Acquisition Parameters
Date_              20131008
Time                  13.56
INSTRUM              DRX400
PROBHD    5 mm SEF 19F-1
PULPROG                zg30
TD                   262144
SOLVENT                DMSO
NS                       16
DS                        2
SW                 120.4695 ppm
O1P                  50.000 ppm
FIDRES             0.173002 Hz
AQ                2.8901875 sec
RG                    812.7
DW                   11.025 usec
TE                    296.2 K
D1               1.00000000 sec
MCREST    0 sec
MCWRK            0.01500000 sec

======== CHANNEL f1 ========
NUC1                    19F
P1                    10.10 usec
PL1       0 dB
SFO1        376.4561069 MHz

F2 - Processing parameters
SI                   131072
SF          376.4371579 MHz
WDW                      EM
SSB       0
LB        0 Hz
GB        0
PC                     4.00
```

^{19}F NMR (376 MHz, DMSO-d$_6$) spectrum of **5.**

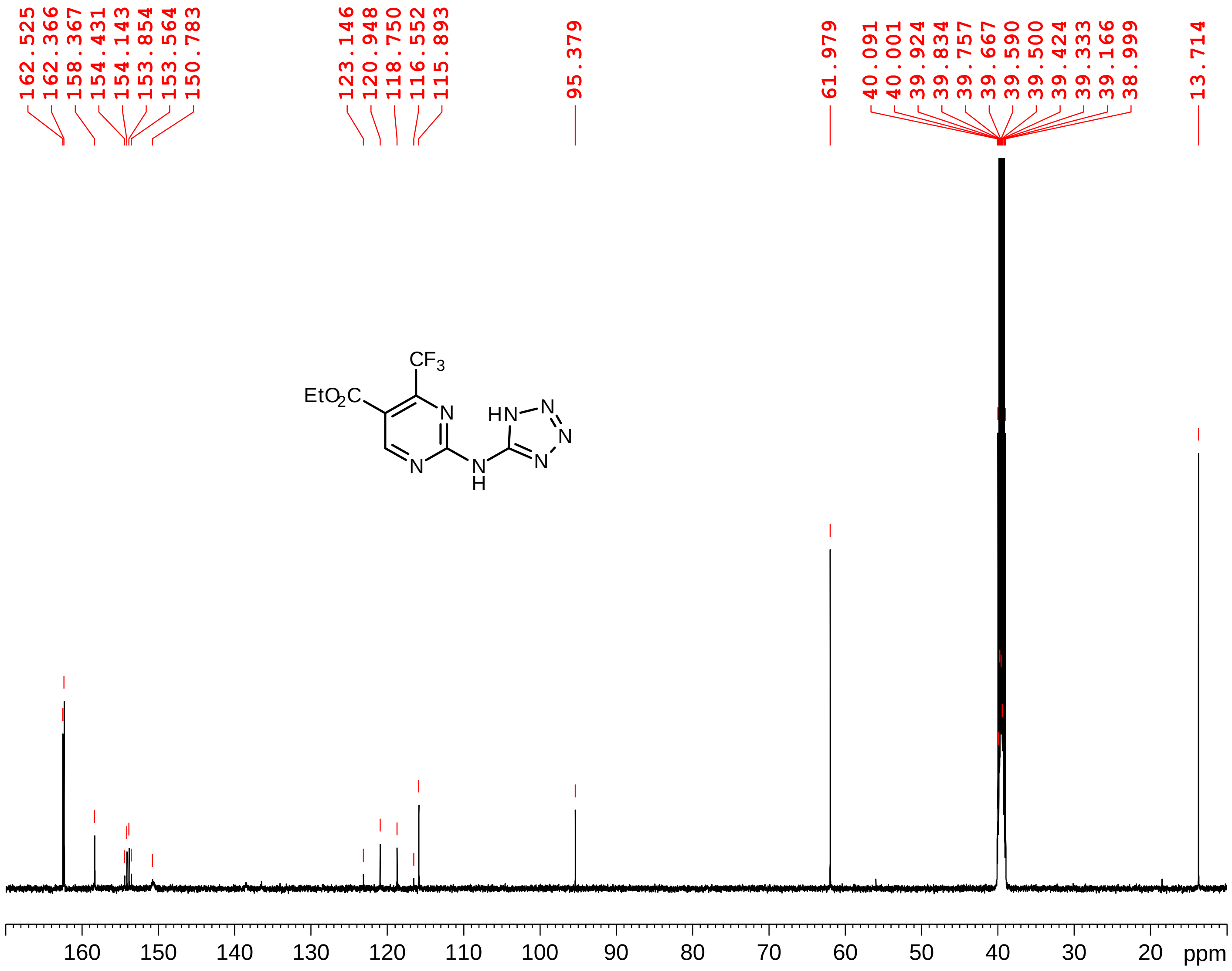


```
Current Data Parameters
NAME              PMV531
EXPNO                 13
PROCNO                 1

F2 - Acquisition Parameters
Date_           20131008
Time               14.11
INSTRUM            AV500
PROBHD    5 mm PABBO BB-
PULPROG          zgpg30
SOLVENT             DMSO
TD                 65536
SW              200.7828 ppm
O1P              100.000 ppm
FIDRES          0.385323 Hz
NS                  4096
DS                     8
AQ             1.2976629 sec
RG                   203
TE                 297.7 K
D1            1.00000000 sec
D11           0.03000000 sec
TD0                    1

======== CHANNEL f1 ========
NUC1                 13C
P1                  9.00 usec
PL1       0 dB
PL1W        115.29558563 W
SFO1         125.7703648 MHz

======== CHANNEL f2 ========
CPDPRG2          waltz16
NUC2                  1H
PCPD2              75.00 usec
PL2               120.00 dB
PL12               17.00 dB
PL13               20.00 dB
PL2W      0 W
PL12W         0.40445811 W
PL13W         0.20270923 W
SFO2         500.1325007 MHz
```

^{13}C NMR (126 MHz, DMSO-d_6) spectrum of **5.**

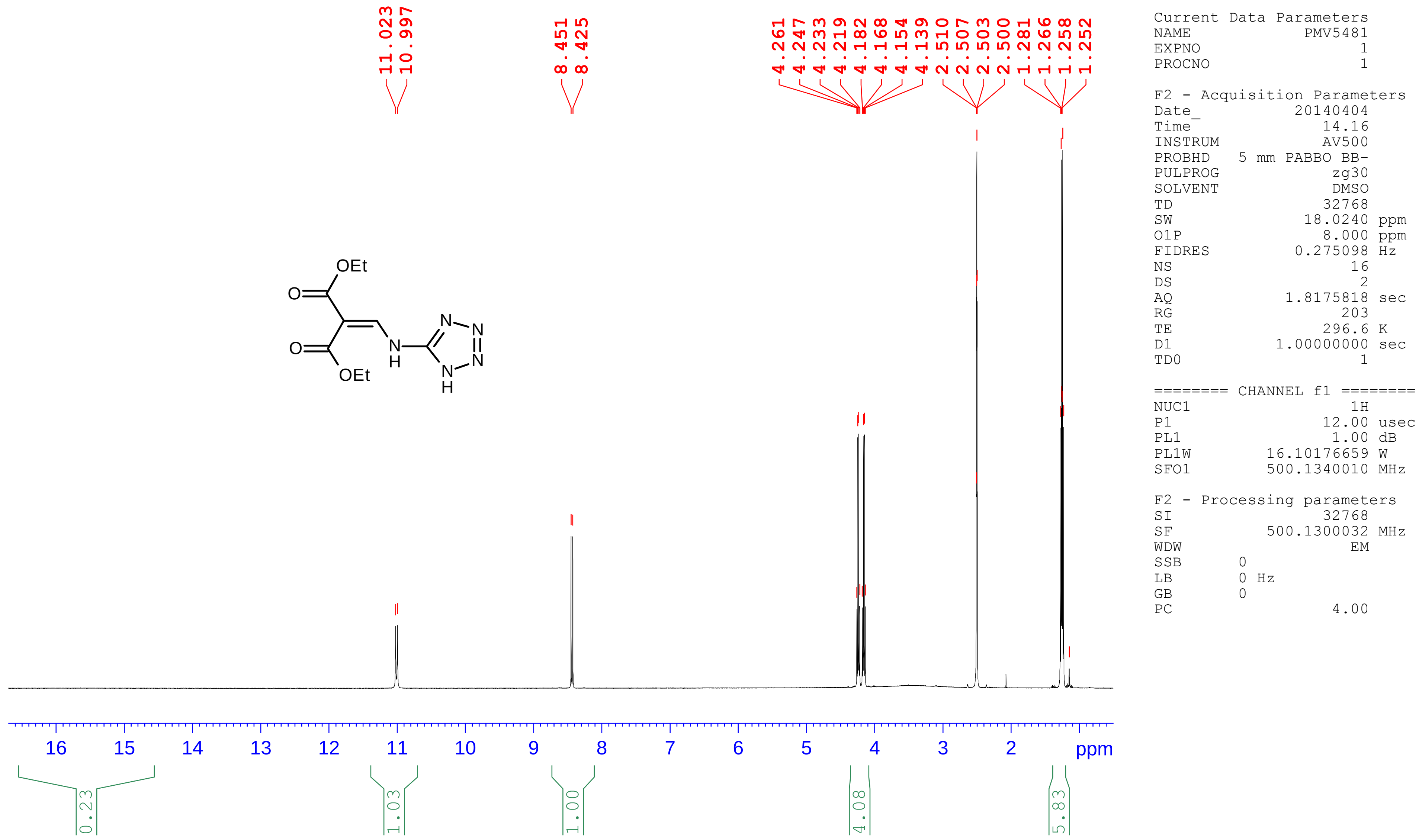

^{1}H NMR (500 MHz, DMSO-d_6) spectrum of **6.**

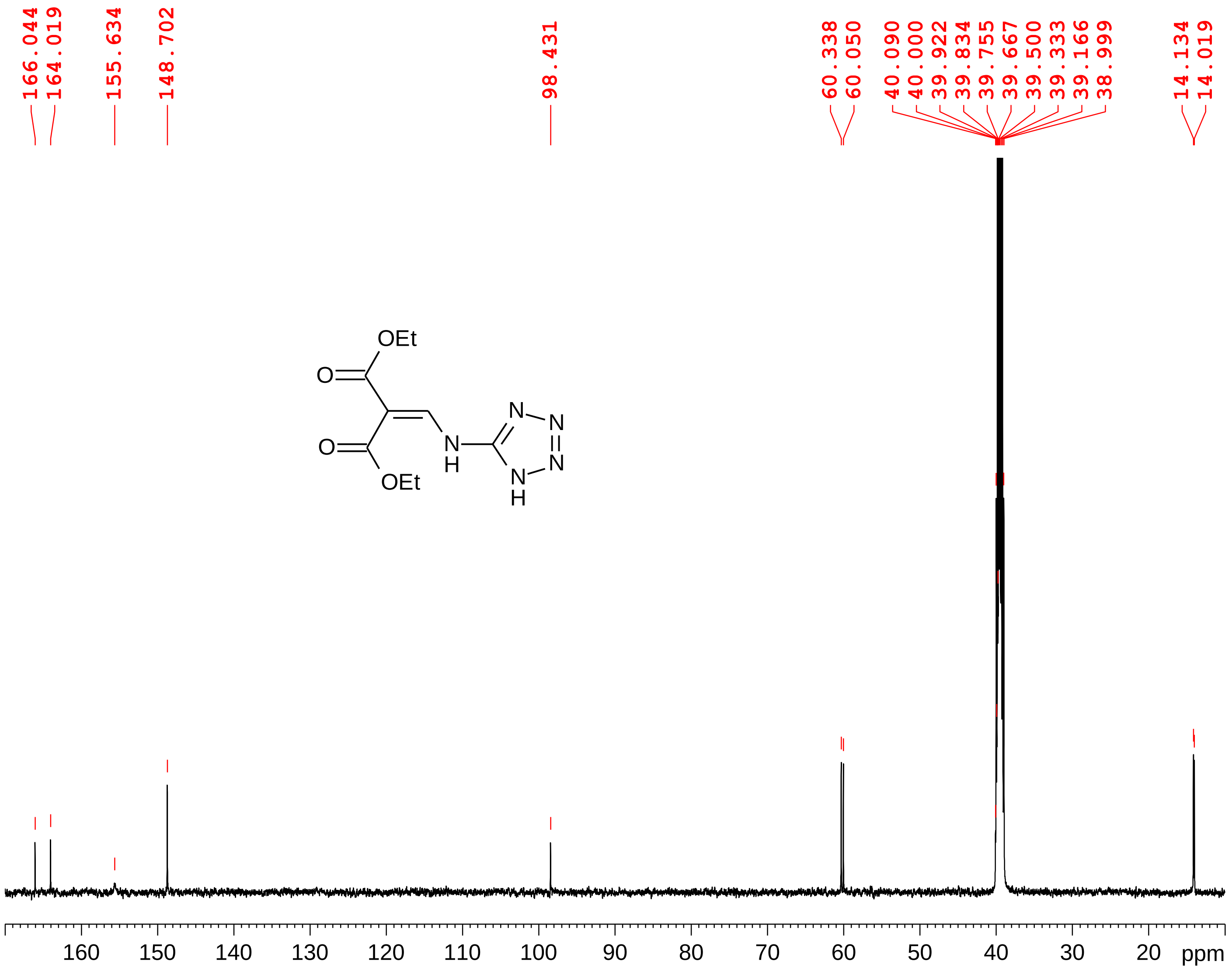


```
Current Data Parameters
NAME              PMV5481
EXPNO                  13
PROCNO                  1

F2 - Acquisition Parameters
Date_            20140404
Time                13.27
INSTRUM             AV500
PROBHD    5 mm PABBO BB-
PULPROG           zgpg30
SOLVENT              DMSO
TD                  32768
SW               200.7828 ppm
O1P               100.000 ppm
FIDRES           0.770646 Hz
NS                   2048
DS                      8
AQ              0.6488564 sec
RG                    203
TE                  296.8 K
D1             0.80000001 sec
D11            0.03000000 sec
TD0                     1

======== CHANNEL f1 ========
NUC1                  13C
P1                   9.00 usec
PL1          0 dB
PL1W         115.29558563 W
SFO1          125.7703648 MHz

======== CHANNEL f2 ========
CPDPRG2           waltz16
NUC2                   1H
PCPD2               75.00 usec
PL2                  1.00 dB
PL12                17.00 dB
PL13                20.00 dB
PL2W          16.10176659 W
PL12W          0.40445811 W
PL13W          0.20270923 W
SFO2          500.1325007 MHz

F2 - Processing parameters
SI                  32768
SF            125.7578520 MHz
WDW                    EM
SSB          0
LB                   3.00 Hz
GB           0
PC                   1.40
```

^{13}C NMR (126 MHz, DMSO-d$_6$) spectrum of **6.**

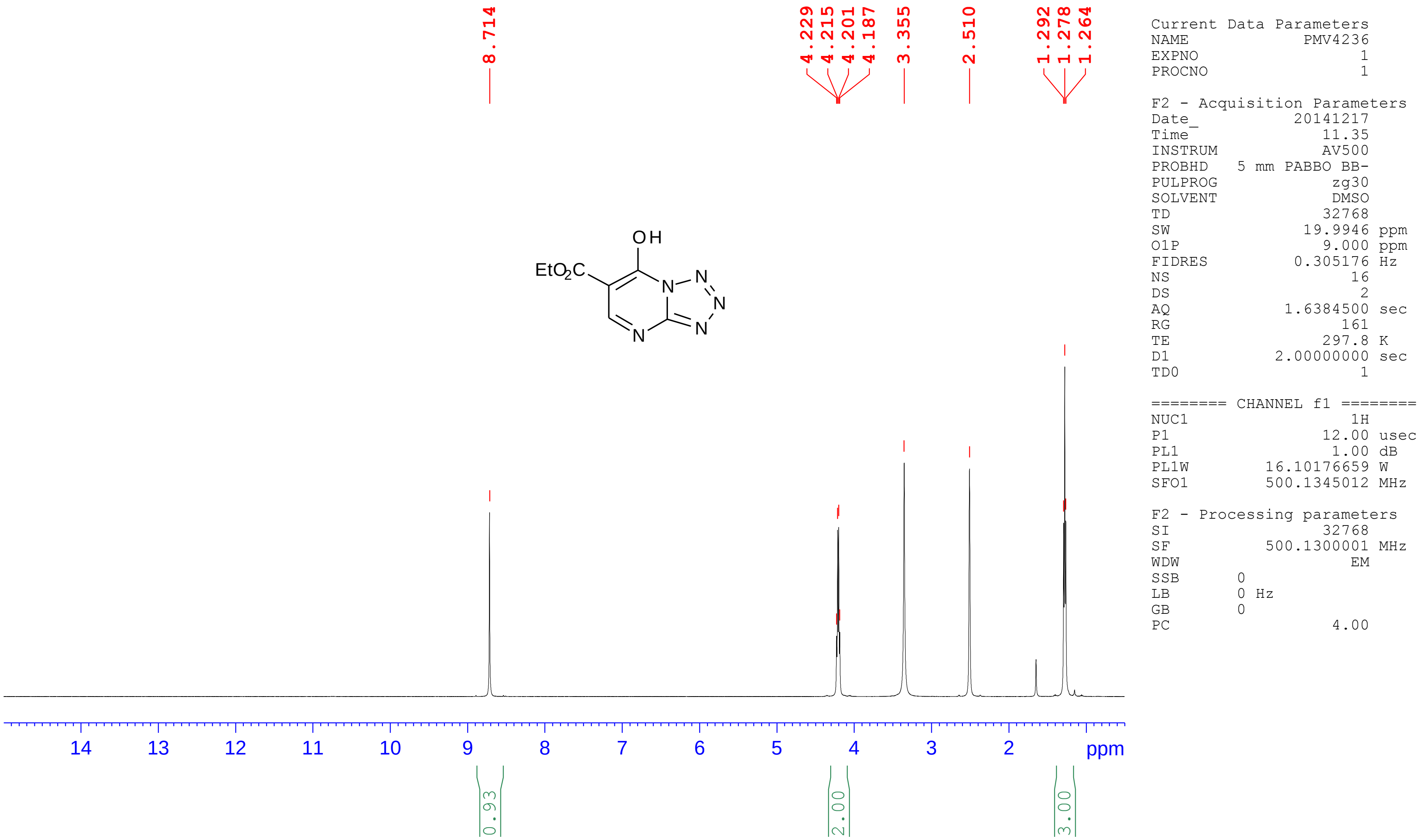

^{1}H NMR (500 MHz, DMSO-d_6) spectrum of **7.**

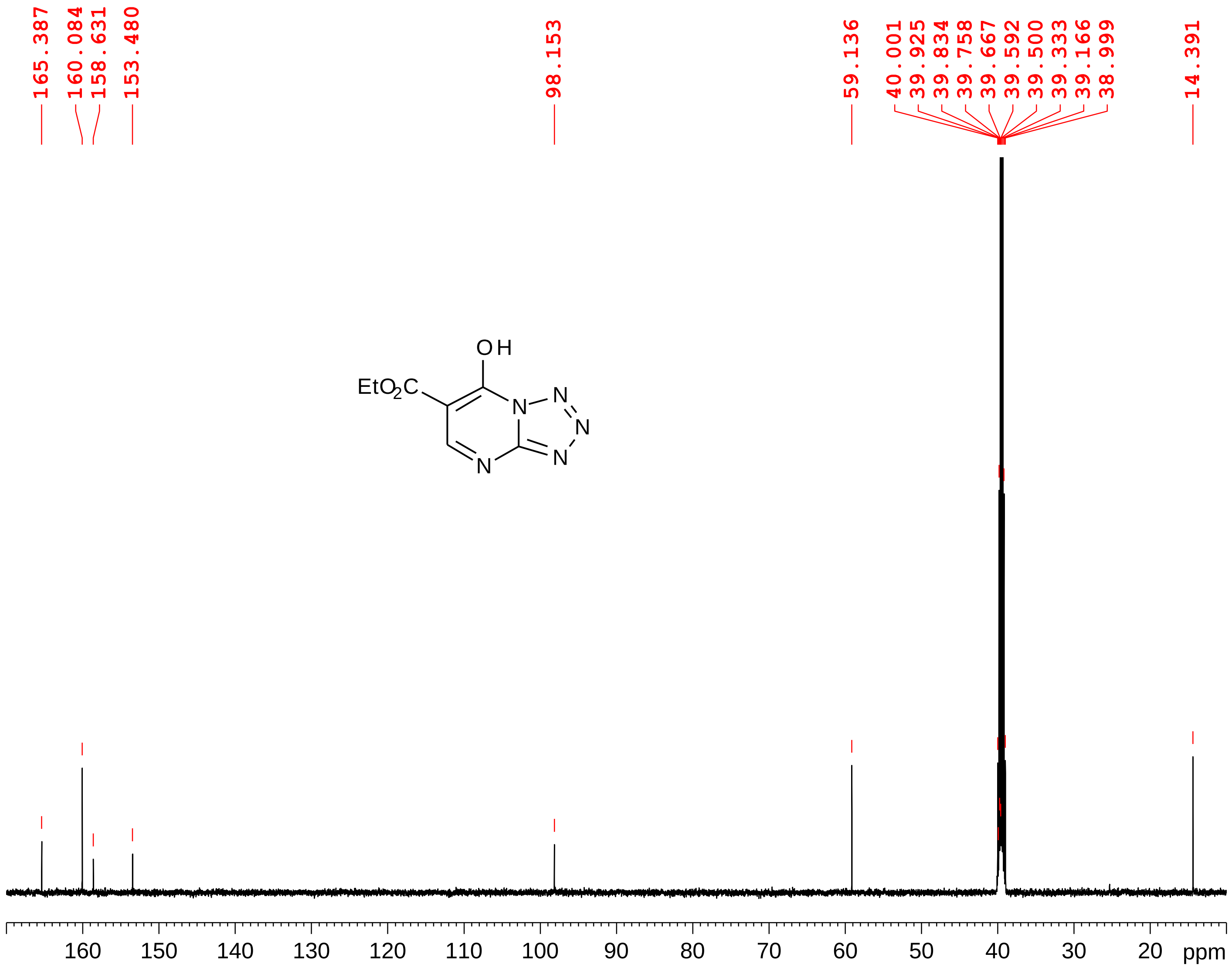


```
Current Data Parameters
NAME              PMV4236
EXPNO                  13
PROCNO                  1

F2 - Acquisition Parameters
Date_            20141222
Time                12.50
INSTRUM             AV500
PROBHD   5 mm PABBO BB-
PULPROG           zgpg30
SOLVENT              DMSO
TD                  32768
SW               200.7828 ppm
O1P               100.000 ppm
FIDRES           0.770646 Hz
NS                    512
DS                      8
AQ              0.6488564 sec
RG                    203
TE                  297.6 K
D1             0.80000001 sec
D11            0.03000000 sec
TD0                     1

======== CHANNEL f1 ========
NUC1                  13C
P1                   9.00 usec
PL1        0 dB
PL1W      115.29558563 W
SFO1      125.7703648 MHz

======== CHANNEL f2 ========
CPDPRG2           waltz16
NUC2                   1H
PCPD2               75.00 usec
PL2               120.00 dB
PL12               17.00 dB
PL13               20.00 dB
PL2W       0 W
PL12W       0.40445811 W
PL13W       0.20270923 W
SFO2      500.1325007 MHz

F2 - Processing parameters
SI                  32768
SF        125.7578506 MHz
WDW                    EM
SSB        0
LB                   1.00 Hz
GB         0
PC                   1.00
```

^{13}C NMR (126 MHz, DMSO-d_6) spectrum of **7.**

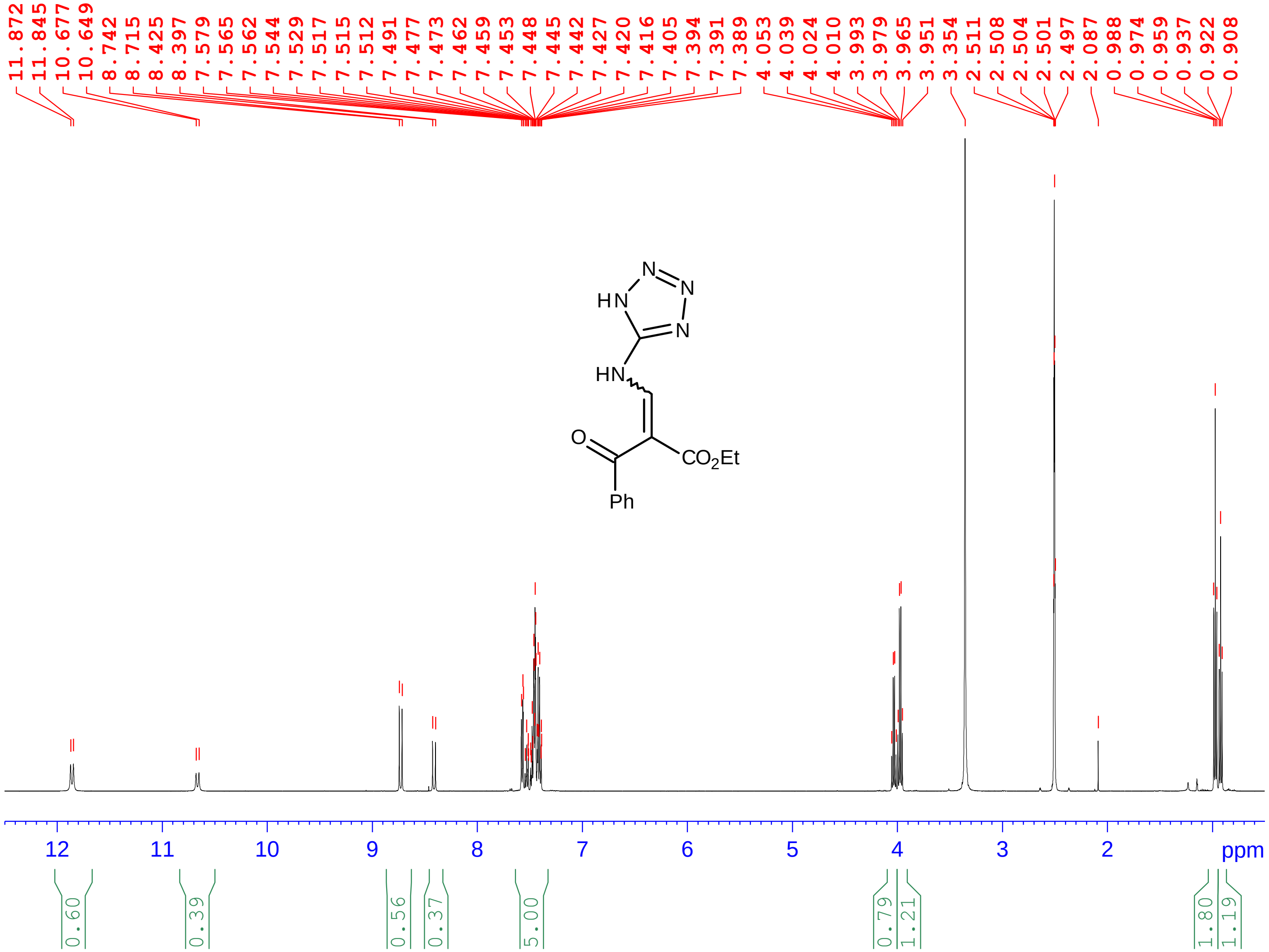


```
Current Data Parameters
NAME              PMV5344
EXPNO                   1
PROCNO                  1

F2 - Acquisition Parameters
Date_            20140508
Time                12.47
INSTRUM             AV500
PROBHD    5 mm PABBO BB-
PULPROG              zg30
SOLVENT              DMSO
TD                  32768
SW               16.0214 ppm
O1P                7.000 ppm
FIDRES          0.244532 Hz
NS                     16
DS                      2
AQ             2.0447731 sec
RG                    144
TE                  296.7 K
D1            1.00000000 sec
TD0                     1

======== CHANNEL f1 ========
NUC1                   1H
P1                  12.00 usec
PL1                  1.00 dB
PL1W          16.10176659 W
SFO1          500.1335009 MHz

F2 - Processing parameters
SI                  32768
SF            500.1300031 MHz
WDW                    EM
SSB       0
LB        0  Hz
GB        0
PC                   4.00
```

^{1}H NMR (500 MHz, DMSO-d$_6$) spectrum of **9.**

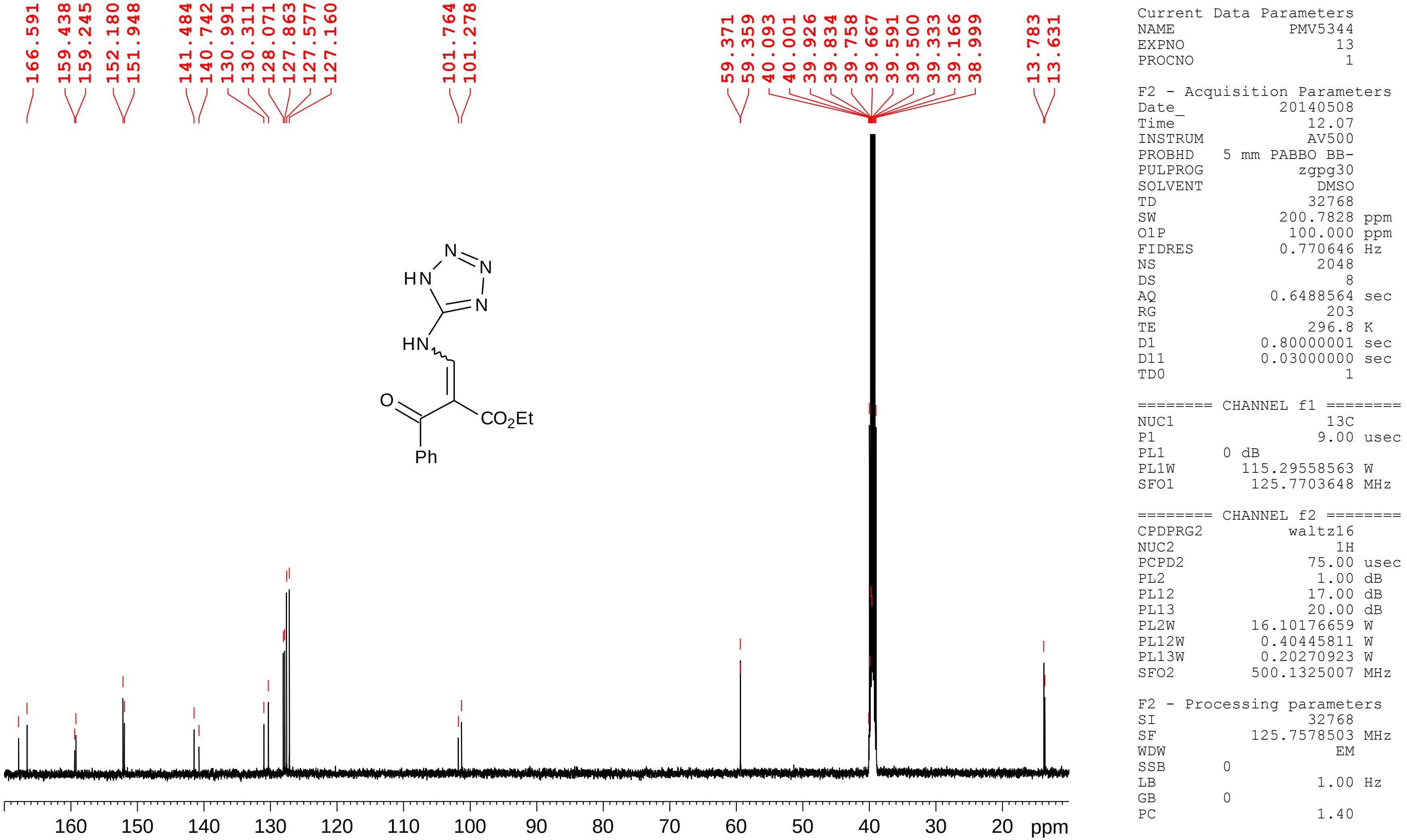

^{13}C NMR (126 MHz, DMSO-d$_6$) spectrum of **9.**

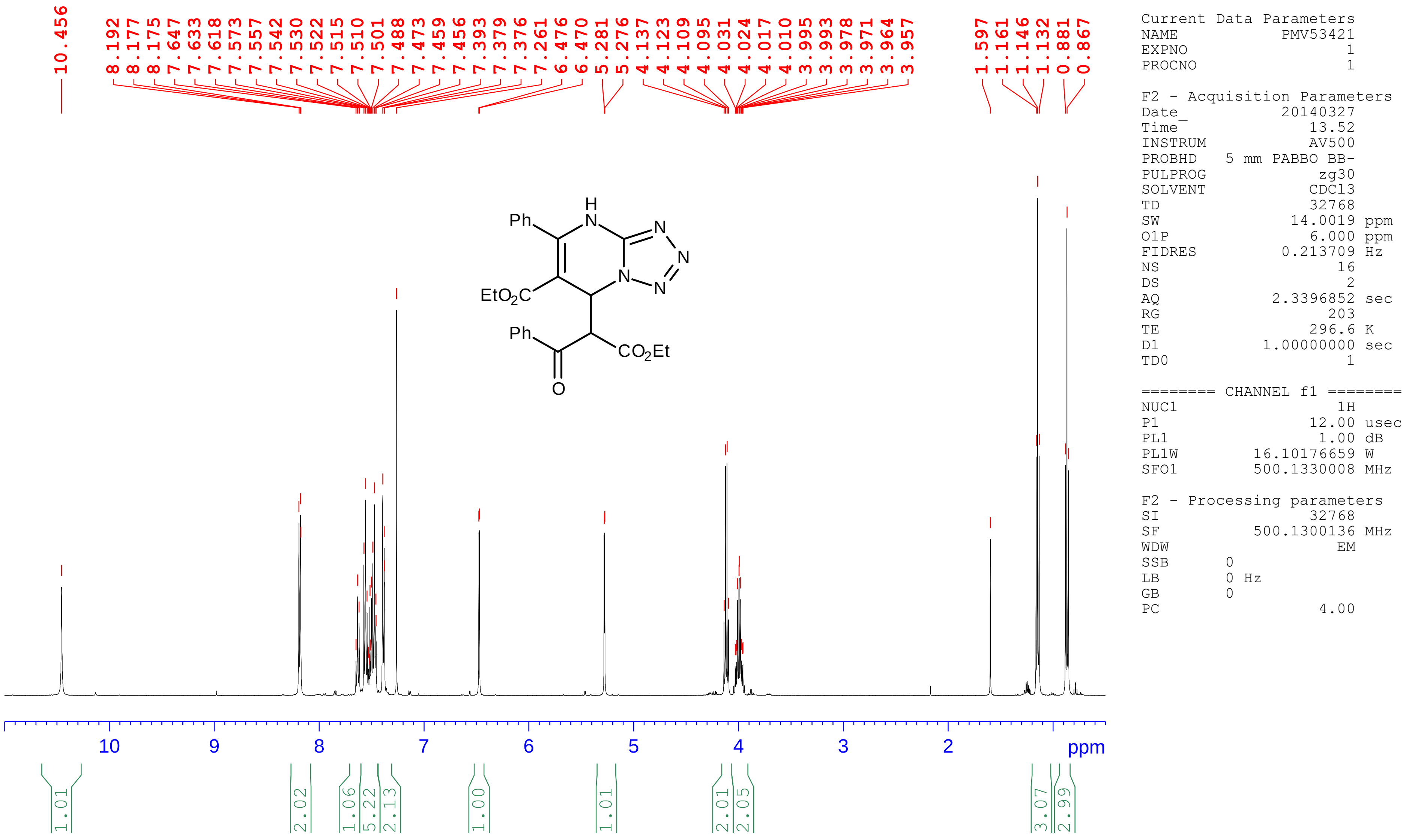

^{1}H NMR (500 MHz, $CDCl_3$) spectrum of **10.**

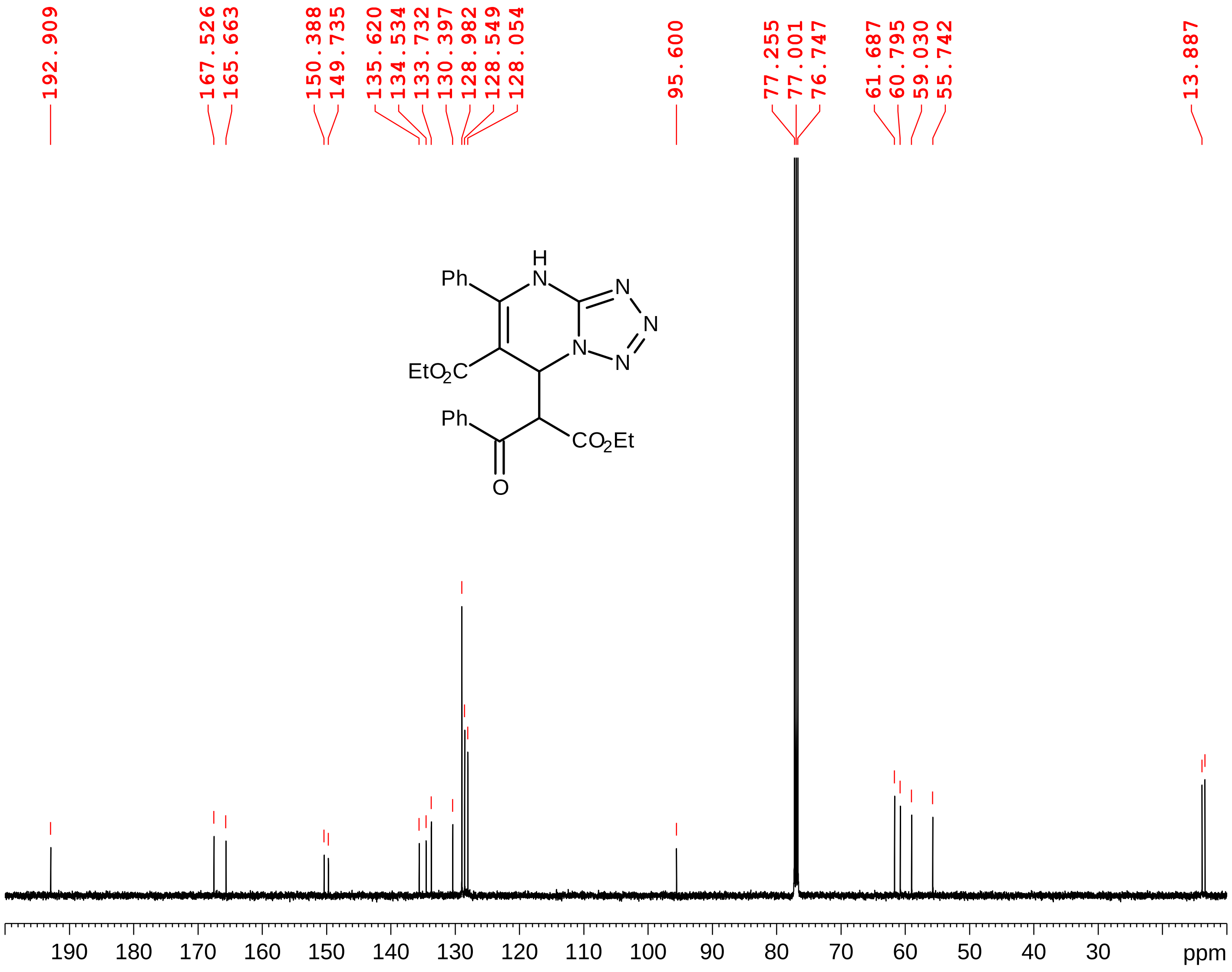

Current Data Parameters
NAME PMV53421
EXPNO 13
PROCNO 1

F2 - Acquisition Parameters
Date_ 20140328
Time 14.03
INSTRUM AV500
PROBHD 5 mm PABBO BB-
PULPROG zgpg30
SOLVENT CDCl3
TD 65536
SW 219.2360 ppm
O1P 105.000 ppm
FIDRES 0.420739 Hz
NS 1024
DS 8
AQ 1.1884362 sec
RG 203
TE 297.6 K
D1 0.80000001 sec
D11 0.03000000 sec
TD0 1

======== CHANNEL f1 ========
NUC1 13C
P1 9.00 usec
PL1 0 dB
PL1W 115.29558563 W
SFO1 125.7709936 MHz

======== CHANNEL f2 ========
CPDPRG2 waltz16
NUC2 1H
PCPD2 75.00 usec
PL2 1.00 dB
PL12 17.00 dB
PL13 20.00 dB
PL2W 16.10176659 W
PL12W 0.40445811 W
PL13W 0.20270923 W
SFO2 500.1325007 MHz

F2 - Processing parameters
SI 65536
SF 125.7577918 MHz
WDW EM
SSB 0
LB 1.00 Hz
GB 0
PC 1.40

^{13}C NMR (126 MHz, $CDCl_3$) spectrum of **10.**

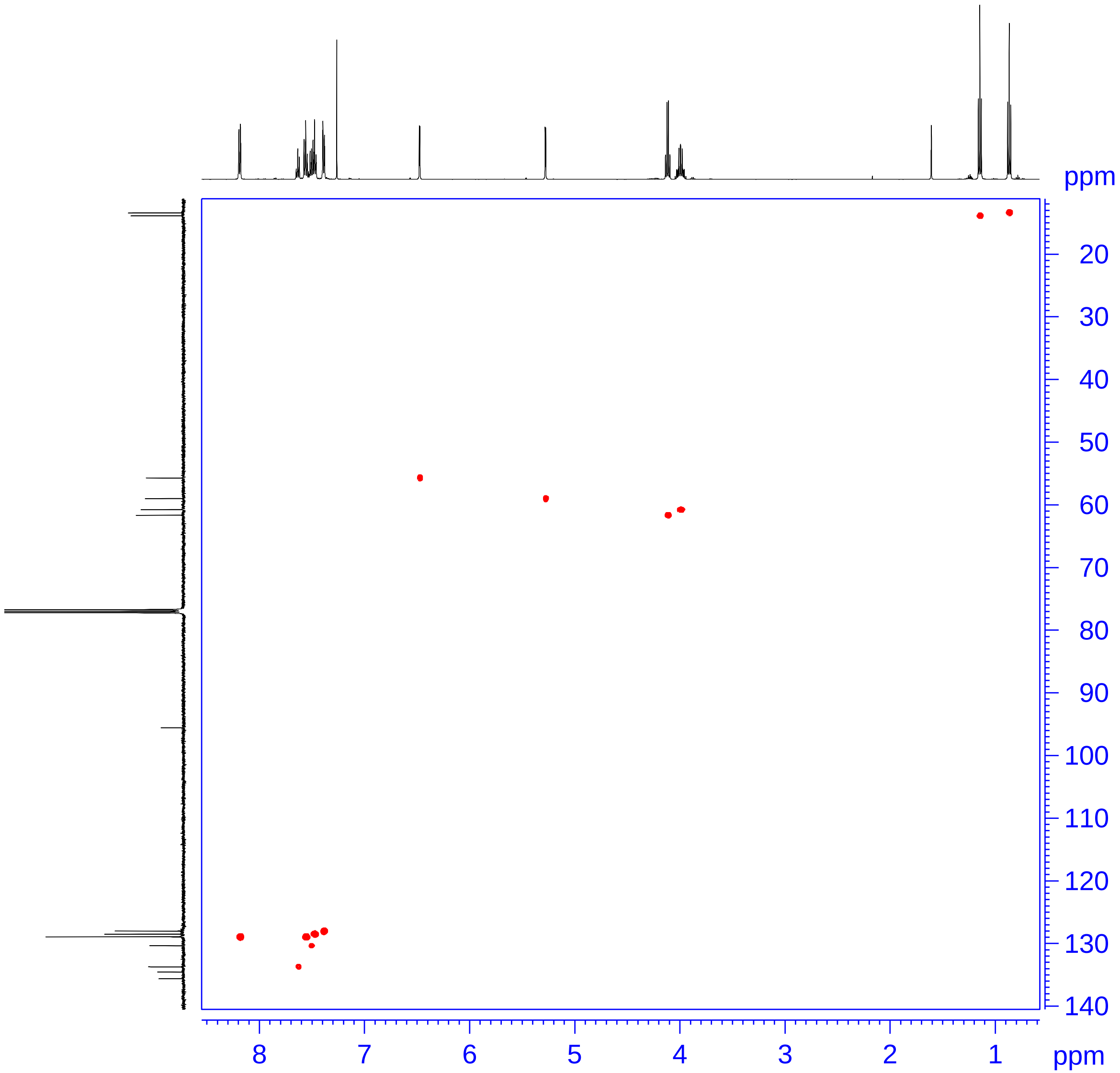

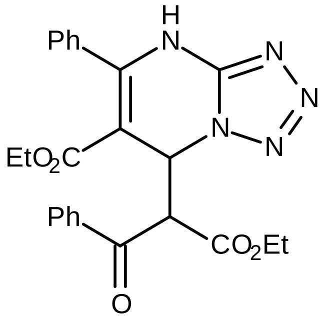

2D ^{1}H–^{13}C HSQC (500 MHz, $CDCl_3$) spectrum of **10.**

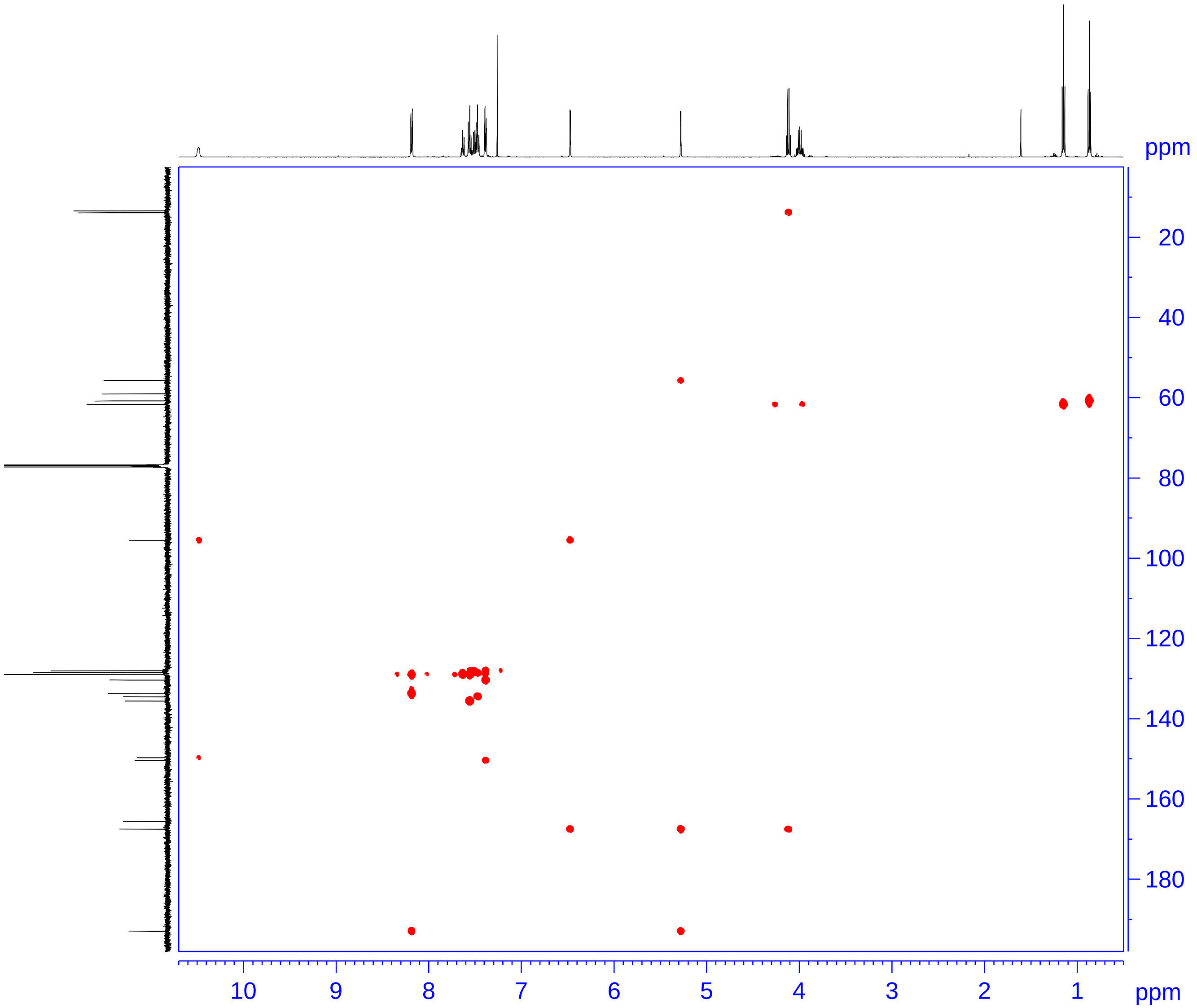

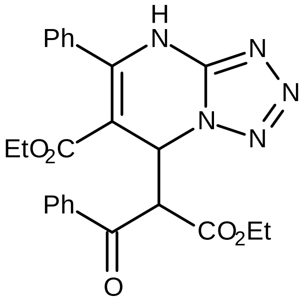

2D ^{1}H–^{13}C HMBC (500 MHz, $CDCl_3$) spectrum of **10.**

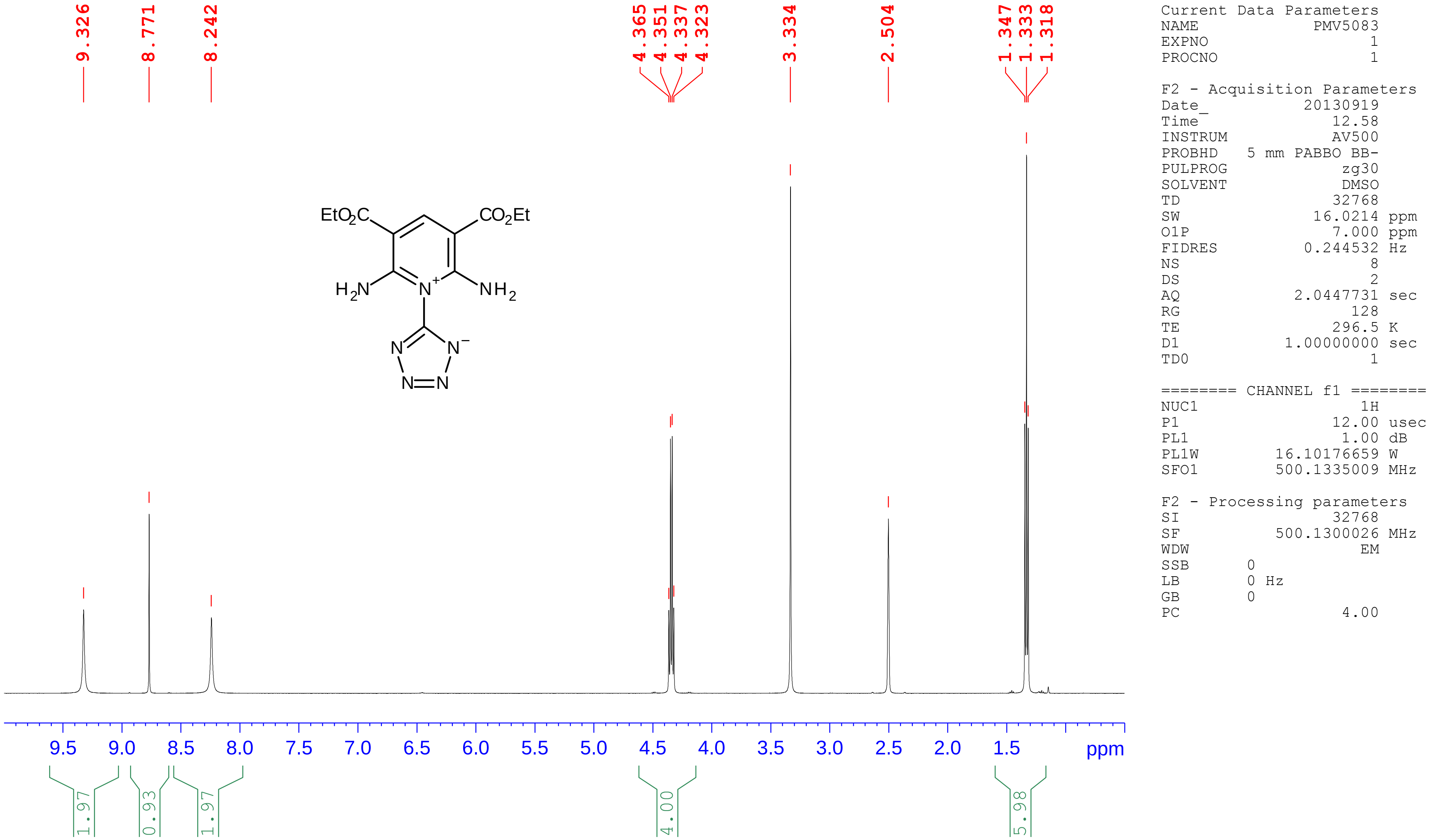

^{1}H NMR (500 MHz, DMSO-d_6) spectrum of **11.**

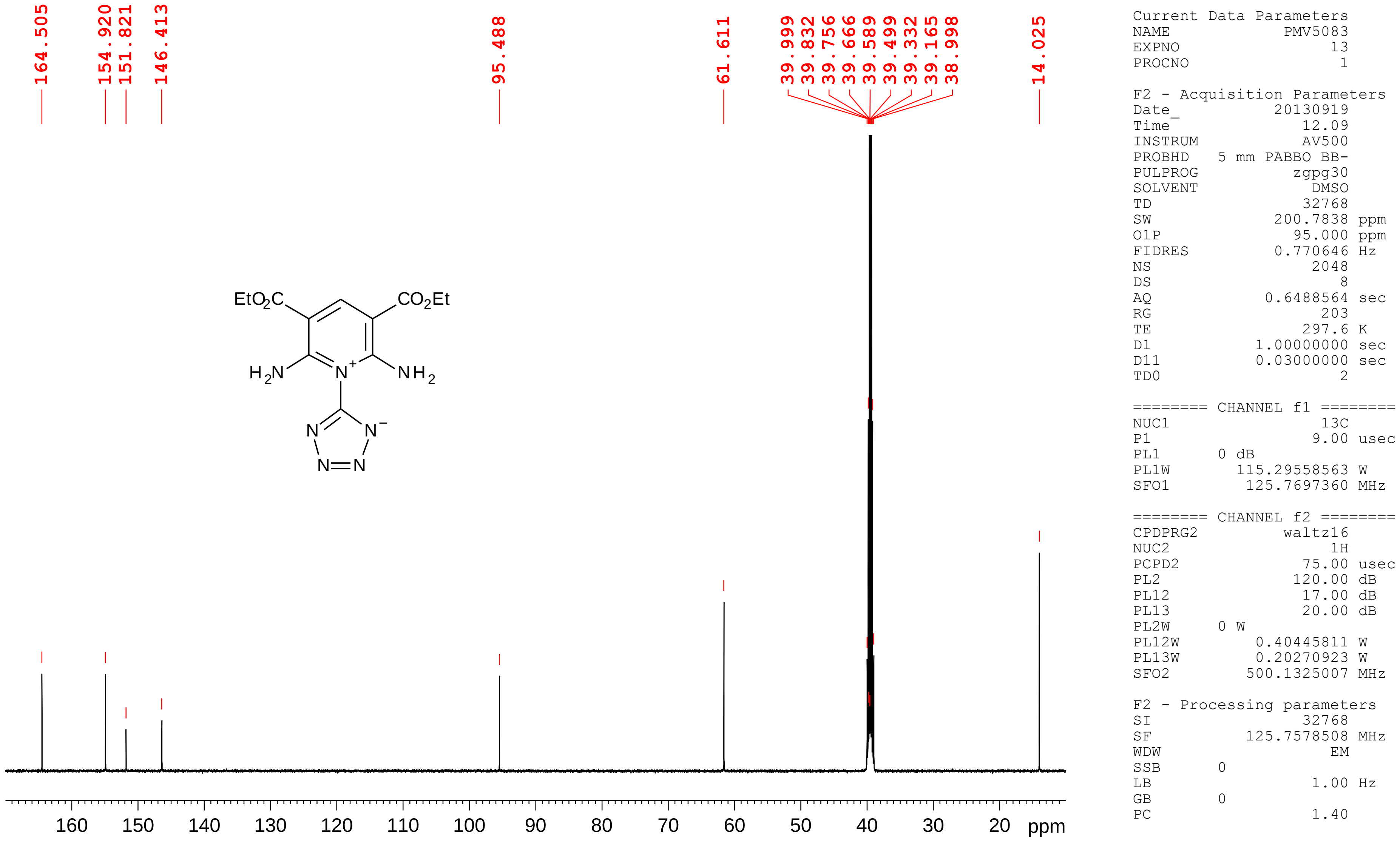

^{13}C NMR (126 MHz, DMSO-d_6) spectrum of **11.**

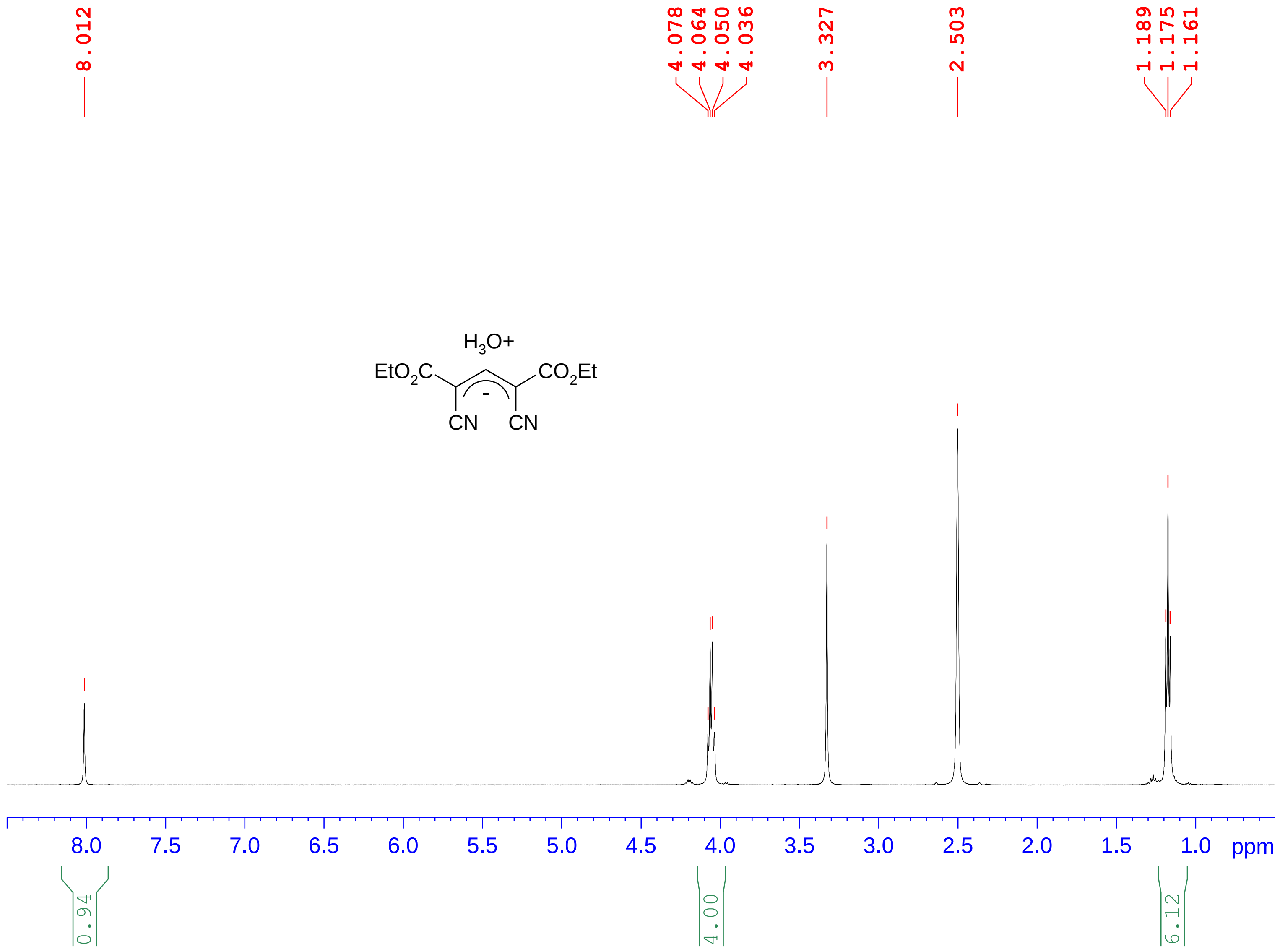


```
Current Data Parameters
NAME              PMV5791
EXPNO                   1
PROCNO                  1

F2 - Acquisition Parameters
Date_            20150121
Time                19.24
INSTRUM             AV500
PROBHD   5 mm PABBO BB-
PULPROG              zg30
SOLVENT              DMSO
TD                  32768
SW               14.0019 ppm
O1P                6.000 ppm
FIDRES          0.213709 Hz
NS                     16
DS                      2
AQ             2.3396852 sec
RG                    203
TE                  296.5 K
D1            1.00000000 sec
TD0                     1

======== CHANNEL f1 ========
NUC1                   1H
P1                  12.00 usec
PL1                  1.00 dB
PL1W          16.10176659 W
SFO1          500.1330008 MHz

F2 - Processing parameters
SI                  32768
SF            500.1300045 MHz
WDW                    EM
SSB      0
LB       0 Hz
GB       0
PC                   4.00
```

11

^{1}H NMR (500 MHz, DMSO-d_6) spectrum of **14.**

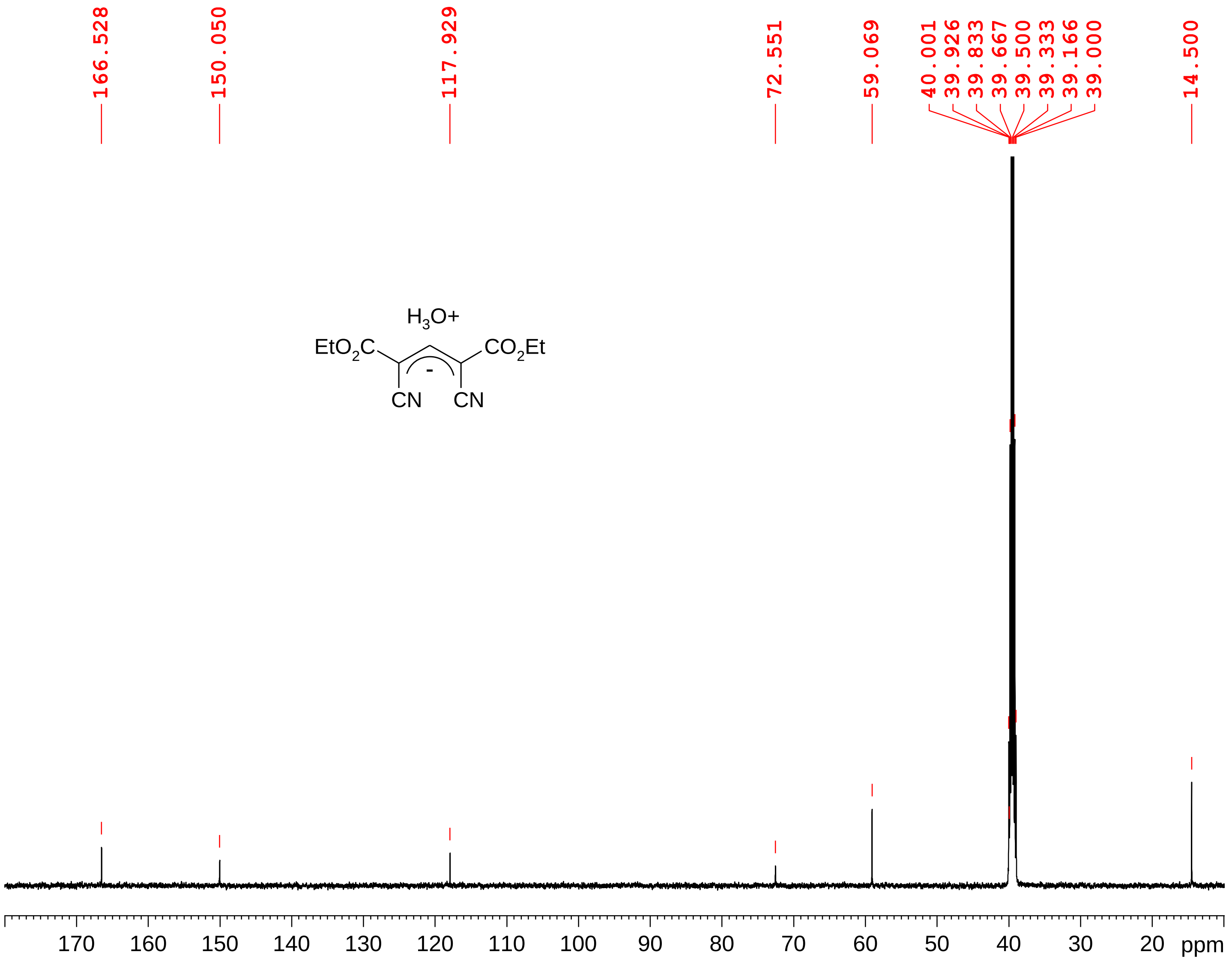


```
Current Data Parameters
NAME              PMV5791
EXPNO                  13
PROCNO                  1

F2 - Acquisition Parameters
Date_            20150121
Time                19.49
INSTRUM             AV500
PROBHD   5 mm PABBO BB-
PULPROG           zgpg30
SOLVENT              DMSO
TD                  32768
SW               219.2349 ppm
O1P               110.000 ppm
FIDRES           0.841477 Hz
NS                   1024
DS                      8
AQ              0.5942430 sec
RG                    203
TE                  297.3 K
D1             0.80000001 sec
D11            0.03000000 sec
TD0                     1

======== CHANNEL f1 ========
NUC1                  13C
P1                   9.00 usec
PL1         0 dB
PL1W         115.29558563 W
SFO1          125.7716224 MHz

======== CHANNEL f2 ========
CPDPRG2           waltz16
NUC2                   1H
PCPD2               75.00 usec
PL2                120.00 dB
PL12                17.00 dB
PL13                20.00 dB
PL2W        0 W
PL12W          0.40445811 W
PL13W          0.20270923 W
SFO2          500.1325007 MHz

F2 - Processing parameters
SI                  65536
SF            125.7578563 MHz
WDW                    EM
SSB         0
LB                   2.00 Hz
GB          0
PC                   1.40
```

^{13}C NMR (126 MHz, DMSO-d_6) spectrum of **14.**
